# Winner-take-all in a phase oscillator system with adaptation

**DOI:** 10.1038/s41598-017-18666-3

**Published:** 2018-01-11

**Authors:** Oleksandr Burylko, Yakov Kazanovich, Roman Borisyuk

**Affiliations:** 1 Institute of Mathematics, National Academy of Sciences of Ukraine, Tereshchenkivska 3, 01601 Kyiv, Ukraine; 2 Institute of Mathematical Problems of Biology, The Branch of Keldysh Institute of Applied Mathematics of Russian Academy of Sciences, 142290 Pushchino, Russia; 3 School of Computing and Mathematics, Plymouth University, PL4 8AA Plymouth, United Kingdom

## Abstract

We consider a system of generalized phase oscillators with a central element and radial connections. In contrast to conventional phase oscillators of the Kuramoto type, the dynamic variables in our system include not only the phase of each oscillator but also the natural frequency of the central oscillator, and the connection strengths from the peripheral oscillators to the central oscillator. With appropriate parameter values the system demonstrates winner-take-all behavior in terms of the competition between peripheral oscillators for the synchronization with the central oscillator. Conditions for the winner-take-all regime are derived for stationary and non-stationary types of system dynamics. Bifurcation analysis of the transition from stationary to non-stationary winner-take-all dynamics is presented. A new bifurcation type called a Saddle Node on Invariant Torus (SNIT) bifurcation was observed and is described in detail. Computer simulations of the system allow an optimal choice of parameters for winner-take-all implementation.

## Introduction

Winner-take-all (WTA) is a computational principle used in artificial neural networks to implement such functions as competitive learning, decision making, and action selection^1,2^. According to this principle the neurons in the system compete with each other for activation. Typically, only the neuron or neural population with the highest activity elicited by the strongest input wins the competition, inhibiting the other neurons to quiescence.

WTA models can be subdivided into several categories depending on the type of the units used in their construction. Early versions of WTA models used nonspiking units which operated with analog input and output signals^3–6^. Later the functional principles of these models were improved and analytical and computational results were obtained on their dynamics and stability^7–13^. Such systems can be conveniently implemented in VLSI circuits^14–24^. To make WTA systems compatible with brain-like devices, WTA networks of spiking units were developed and studied^25–30^ together with their VLSI counterparts^31–33^. WTA systems were applied for building a silicon retina^34,35^, hierarchical models of vision^36^, and for modeling cognitive functions^37–45^. It has been proven that the winner-take-all operation is computationally powerful compared to other nonlinear operations, such as thresholding^46,47^.

The temporal correlation hypothesis^48^ stimulated the development of WTA systems based on synchronization of oscillatory activity. A system of Van der Pol oscillators with global inhibitory neurons has been developed for consecutive selection of objects in an image^49^, with the WTA regime as a special case. A similar WTA implementation was realized in a system of FitzHugh-Nagumo oscillators with a global inhibitory neuron^50^. In the paper^51^ a system is built from adaptively coupled WTA circuits, where each circuit is an oscillator driven by the interaction between multiple excitatory units and a common inhibitory unit. The competition between excitatory units results in one population of excitatory neurons being active at a time. Switching between the states is controlled by external stimulation.

Traditionally in WTA systems outputs compete for activation via lateral inhibition or recurrent inhibition. In this paper we suggest an alternative approach to the WTA problem based on synchronization in an oscillatory network with a central unit. Consider a system built from oscillators with a radial connection architecture. This means that there is a central oscillator (CO) in the system that is connected with a set of so-called peripheral oscillators (POs) by feedforward and feedback connections. We will show that competition between POs for the synchronization with the CO can be organized in such a way that only one PO can win the competition. This PO will work coherently with the CO while other POs will be out of phase with the CO. This results in a resonant increase of the activity of the winning PO while the activity of the other POs will be reduced to a low level.

We use generalized phase oscillators as the elements of the WTA system. Phase oscillators of the Kuramoto type^52^ have been widely used for describing dynamics of Josephson-junction arrays, neutrino flavor oscillations, semiconductor laser arrays, coupled magnetic systems, and neural networks. Reviews of the mathematical theory of phase oscillator systems and their applications can be found in the publications^53–56^. Systems with phase oscillators and radial connection architectures have been studied in the papers^57–63^. Conventionally, a phase oscillator is described by a single variable, its phase. The natural frequencies of the oscillators and coupling strengths are the parameters of the system.

Generalized phase oscillators differ from original Kuramoto oscillators by the transformation of some parameters of the system into dynamical variables. In the case of the system considered here the variables include the natural frequency of the CO and the strengths of connections from POs to the CO. The natural frequency of the CO is adapted in the direction of its current value. The connection strength of a PO is adapted as a function of the similarity between its phase and the phase of the CO. To obtain the WTA regime the connection strengths from POs to the CO should be positive and the connection strengths from the CO to POs should be negative.

The advantage of our WTA phase oscillator system is the possibility of implementing it in hardware as a laser optical device or as a Josephson junction array. Note that there are no connections between POs, so the whole number of connections in the system is 2*n*.

WTA systems of generalized phase oscillators with a central element have previously been applied to attention modeling and visual search^64,65^. Here we present for the first time a rigorous mathematical analysis of their dynamics.

We develop a new mathematical theory of WTA oscillatory networks. In the case of POs with identical natural frequencies we derive conditions when only one PO wins the competition. This winner is synchronized with the CO, while all other POs are in antiphase to the CO. These WTA dynamics correspond to the existence of a stable equilibrium in the phase space of the system.

Using perturbation theory and bifurcation analysis, we demonstrate that in the case of non-identical natural frequencies of POs the system can demonstrate both stable and oscillatory versions of the WTA regime depending on the relations between the parameters of the system. In the stable case, the winning PO works nearly inphase with the CO, while the absolute values of the difference between the phases of other POs and the CO exceed *π*/2. In the oscillatory version of WTA there is a single winner which works nearly inphase with the CO, while the phases of other POs oscillate far from the phase of the CO or run indefinitely in the positive or negative direction. We show that the appearance of the oscillatory WTA regime is due to a Saddle Node on Invariant Torus (SNIT) bifurcation which is a generalization of the well-known Saddle Node on Invariant Circle (SNIC) bifurcation (see^66,67^). Note that the SNIT bifurcation of two cycles on a 2–dimensional torus was studied among others by C. Baesens *et al*.^68,69^. The same type of bifurcations has been addressed in considerable detail for the 3-dimensional case, though without the reinjection, by A. Chenciner^70–72^ and C. Baesens and R. S. MacKay^73^.

Finally, using massive simulations of the model we clarify how the parameters of the system affect the results of WTA.

## Model Formulation

The system that we consider contains a central oscillator (CO) and a set of *n* peripheral oscillators (POs). The CO interacts with POs through feedforward and feedback connections. There are no lateral connections between POs. The dynamics of the system are described by the following equations

1 $$\frac{d{\theta }_{0}}{dt}={\omega }_{0}+\frac{1}{n}\sum _{j\mathrm{=1}}^{n}{a}_{j}\,f({\theta }_{j}-{\theta }_{0}),$$

2 $$\frac{d{\theta }_{i}}{dt}={\omega }_{i}+bg({\theta }_{0}-{\theta }_{i}),\quad i=1,\ldots ,n,$$

3 $$\frac{d{\omega }_{0}}{dt}=\frac{\alpha }{n}\sum _{j\mathrm{=1}}^{n}{a}_{j}\,f({\theta }_{j}-{\theta }_{0}),$$

4 $$\frac{d{a}_{i}}{dt}=\beta (-{a}_{i}+c+\gamma h({\theta }_{i}-{\theta }_{0})),\quad i=1,\ldots ,n,$$

where $({\theta }_{0},{\theta }_{1},\ldots ,{\theta }_{n},{\omega }_{0},{a}_{1},\ldots {a}_{n})\in {{\mathbb{R}}}^{n+1}\times {\mathbb{R}}\times {{\mathbb{R}}}^{n}$ are the variables, *ω*_*i*_, *i* = 1, …, *n*, *α*, *β*, *γ*, *b*, *c* are the parameters, In (1)–(4) *θ*_*i*_ are the current phases of the oscillators, *ω*_*i*_ are the natural frequencies of the oscillators, *a*_*i*_ and *b* are the connection strengths between the oscillators. We will also associate *a*_*i*_ with the amplitudes of oscillations of POs. Positive values of connection strengths correspond to synchronizing interaction and negative values correspond to desynchronizing interaction. We always suppose that *α*, *β*, *γ*, *c* > 0.

The meaning of equations (1)–(4) will be explained a bit below. Right now let us introduce the restrictions on the functions used in these equations. The functions *f*, *g*, *h* are assumed to be 2*π*–periodic and satisfy the following conditions

5 $$f(x)=-f(-x),\quad f^{\prime} \mathrm{(0)} > \mathrm{0,}\quad f^{\prime} (\pi )=\mathrm{0,}$$

6 $$g(x)=-g(-x),\quad g^{\prime} \mathrm{(0)} > \mathrm{0,}\quad g^{\prime} (\pi ) < \mathrm{0,}$$

7 $$h(x)=h(-x),\quad h\mathrm{(0)}=1,\quad h(\pi )=\mathrm{0,}\quad h^{\prime} \mathrm{(0)}=h^{\prime} (\pi )=0.$$

Thus the functions *f* and *g* are odd and the function *h* is even. Periodicity and oddness of the functions *f* and *g* imply the conditions *f*(0) = *f*(*π*) = *g*(0) = *g*(*π*) = 0. We assume that the functions *f*(*x*), *g*(*x*) do not have other zeros in the interval (0,*π*). We also require that *h*(*x*) is monotonic on [0, *π*] (and [−*π*, 0]).

Equations (1)–(4) can be considered as a generalization of standard Kuramoto equations for phase oscillators^52^ by introducing in addition to two phase equations an equation for the adaptation of the natural frequency of the CO *ω*_0_ and the equations for the adaptation of the amplitudes *a*_*i*_. The meaning of equation (3) becomes clear if it is rewritten as^64^

$$\frac{d{\omega }_{0}}{dt}=\alpha (\frac{d{\theta }_{0}}{dt}-{\omega }_{0}).$$

According to this equation the natural frequency of the CO is adapted in the direction of the current frequency. The parameter *α* controls the speed of adaptation.

The meaning of equation (4) is that there is resonant increase of the amplitude of oscillations of the *i*-th PO to the level *c* + *γ* if this PO works inphase with the CO, otherwise this amplitude decreases to a low level *c*. The parameter *β* controls the speed of amplitude adaptation.

System (1)–(4) is similar to the systems invented in^65^ with the aim to organize a competition between POs for the synchronization with the CO in such a way that in a typical case only one PO can win the competition. We will show that this is possible if *b* < 0. Other types of dynamics including multistable and chaotic regimes are also possible depending on the values of the parameters.

Two types of dynamics can be associated with the WTA regime in system (1)–(4). In the stationary case we say that the *i*-th PO wins the competition if it is the only PO that asymptotically has the amplitude of oscillations equal to *c* + *γ*, while the amplitudes of other POs are equal to *c*. In the non-stationary case the amplitudes of POs are not constants anymore. We say that the *i*-th PO wins the competition if its amplitude is asymptotically concentrated in a region above the value *c* + *γ* − *δ*, while the amplitudes of other POs vary in a region below the value *c* + *δ*, where *δ* is a small number. Our aim is to find conditions when stationary and non-stationary WTA regimes take place. For this reason we investigate the dynamics of system (1)–(4) in two cases, corresponding to identical and non-identical POs.

**Figure 1:**
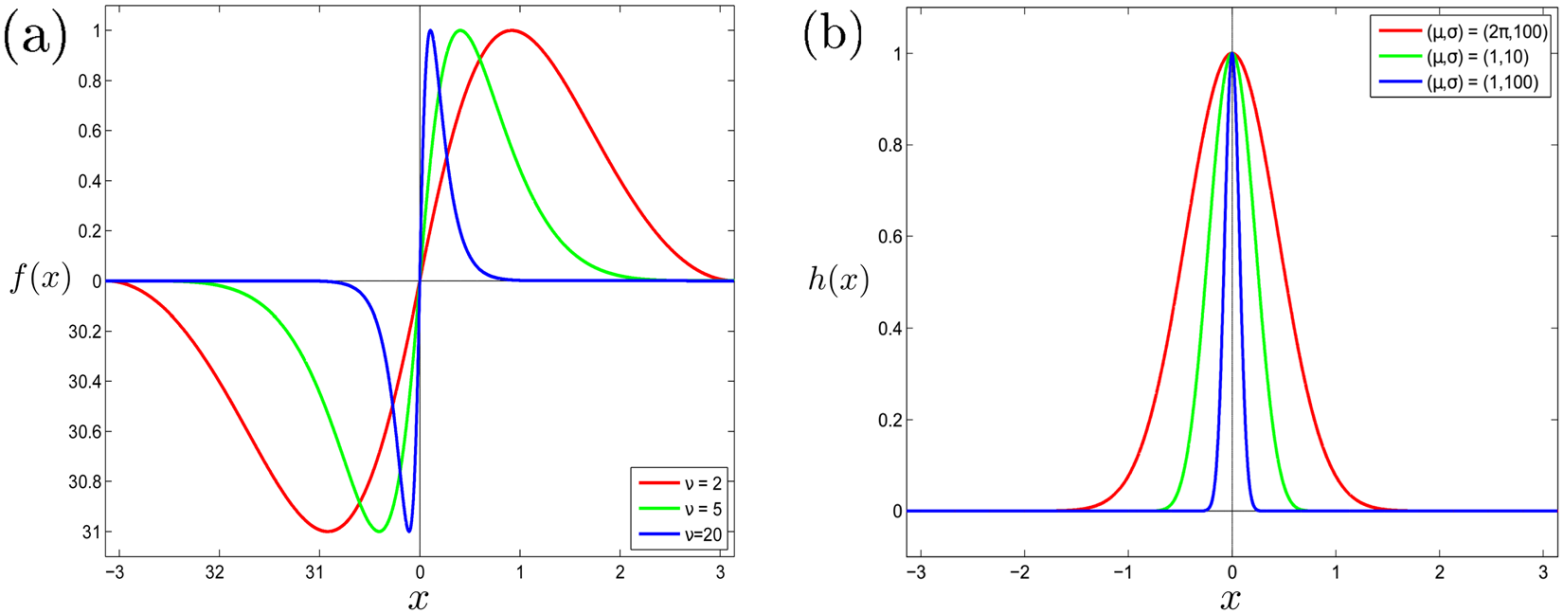
Coupling functions: (**a**) interaction function *f*(*x*) for different *ν*, (**b**) resonance controlling function *h*(*x*) for different *μ*, *σ*.

Some results about the dynamics of (1)–(4) can be obtained for the general form of the functions (5)–(7). More advanced and complicated results demand a specification of the functions *f*, *g*, *h*. In this case the following types of the functions will be used:

8 $$f(x)=\{\begin{array}{cc}\displaystyle \sin (\frac{{|x-\pi |}^{\nu }}{{\pi }^{\nu -1}}),\quad  & x\in [0,\pi ],\\ \displaystyle -\sin (\frac{{|x+\pi |}^{\nu }}{{\pi }^{\nu -1}}),\quad  & x\in [-\pi ,0],\end{array}$$

9 $$g(x)=\sin x.$$

10 $$h(x)=\{\begin{array}{cc}{(\frac{{\mu }^{2}-{x}^{2}}{{\mu }^{2}})}^{\sigma },\quad  & |x| < \mu ,\\ 0,\quad  & \mu \le |x|\le \pi ,\end{array}$$

with the parameters *ν* > 1, $\sigma \gg \mathrm{1,}$ $\mu \in \mathrm{(0,}\,\pi )$ (see Fig. 1).

The function *f*(*x*) has a single maximum in the interval (0, *π*) and a symmetric minimum in (−*π*, 0) such that

$${x}_{max}=-{x}_{min}=\pi (1-{2}^{-1/\nu }),\quad f({x}_{max})=-f({x}_{min})=1$$

and

$$\mathop{\mathrm{lim}}\limits_{\nu \to \infty }\,{x}_{{\rm{\max }}}=0.$$

Thus, increasing the value of the parameter *ν* we can continuously move the location of the maximum (and minimum) point from *π*/2 (and −*π*/2) to zero (Fig. 1(a)). The parameter *ν* also specifies the slope of the function *f* at the zero point:

$$f^{\prime} \mathrm{(0)}=\nu .$$

One can check that *f*′(*π*) = 0 for *ν* > 1, that is the last condition of (5) is satisfied.

The parameter *σ* controls the “width” of the function *h*. Increased values of *σ* make sharper the peak at *h*(0) (Fig. 1(b)). Below we will show how the values of the parameters *ν* and *σ* influence WTA results.

## Results

Using phase differences

11 $${\phi }_{i}={\theta }_{i}-{\theta }_{0},\quad i=1,\ldots ,n,$$

we reduce system (1)–(4) to the equations

12 $$\frac{d{\phi }_{i}}{dt}={\omega }_{i}-{\omega }_{0}-bg({\phi }_{i})-\frac{1}{n}\sum _{i=1}^{n}{a}_{j}\,f({\phi }_{j}),\quad i=1,\ldots ,n,$$

13 $$\frac{d{\omega }_{0}}{dt}=\frac{\alpha }{n}\sum _{i\mathrm{=1}}^{n}{a}_{j}\,f({\phi }_{j}),$$

14 $$\frac{d{a}_{i}}{dt}=\beta (-\,{a}_{i}+c+\gamma h({\phi }_{i})),\quad i=1,\ldots ,n.$$

### Identical peripheral oscillators

Consider a symmetric case of *equal natural frequencies* of POs

15 $${\omega }_{i}=\omega ,\quad i=1,\ldots ,n.$$

Then system (12)–(14) obtains the form

16 $$\begin{array}{cccc}\displaystyle \frac{d{\phi }_{i}}{dt} & = & \displaystyle \omega -{\omega }_{0}-bg({\phi }_{i})-\frac{1}{n}\sum _{i=1}^{n}{a}_{j}f({\phi }_{j}),\, & i=1,\ldots ,n\\ \displaystyle \frac{d{\omega }_{0}}{dt} & = & \displaystyle \frac{\alpha }{n}\sum _{i=1}^{n}{a}_{j}f({\phi }_{j}),\\ \displaystyle \frac{d{a}_{i}}{dt} & = & \displaystyle \beta (-{a}_{i}+c+\gamma h({\phi }_{i})), & i=1,\ldots ,n.\end{array}$$

System (16) has permutation symmetry S_*n*_: the permutation of any two pairs (*φ*_*i*_, *a*_*i*_) and (*φ*_*j*_, *a*_*j*_) does not change the system. We denote

$${\rm{\Phi }}=({\phi }_{1},\ldots ,{\phi }_{n}),\quad a=({a}_{1},\ldots ,{a}_{n}),$$

$${{\rm{\Phi }}}_{k}:=(\mathop{\underbrace{\mathrm{0,}\ldots ,\,0}}\limits_{k},\mathop{\underbrace{\pi ,\ldots \pi }}\limits_{n-k}),\quad k=0,\ldots ,n,$$

$${{\rm{\Psi }}}_{k}=(\mathop{\underbrace{c+\gamma ,\ldots c+\gamma }}\limits_{k},\mathop{\underbrace{c,\ldots c}}\limits_{n-k}),\quad k=\mathrm{0,}\ldots ,n.$$

Using this notation we can formulate the following statement.

#### **Proposition 1.**

*System* (16) *has fixed points*

17 $${P}_{k}=({\rm{\Phi }},\,{\omega }_{0},\,a)=({{\rm{\Phi }}}_{k},\,\omega ,\,{{\rm{\Psi }}}_{k}),\quad k=0,\ldots ,n.$$

Since the functions *f*, *g* do not intersect the abscissa in (0, *π*), system (16) has 2^*n*^ fixed points *P*_*k*_ corresponding to different values of *k*.

For fixed *k* there are ${C}_{n}^{k}$ symmetric points *P*_*k*_ whose multiplication is conditioned by permutation symmetry. The point *P*_1_ corresponds to inphase synchronization of the CO with exactly one PO, while other POs are in antiphase to the CO. This case can be considered as a WTA procedure when one PO wins the competition for the synchronization with the CO. This PO is called *the winner*, while other POs are called *losers*.

The following statement describes stability of fixed points presented in Proposition 1.

#### **Proposition 2.**

*System* (12)–(14) *with identical POs can have either the stable equilibrium P*_*n*_ (*full synchronization*) *for b* > 0 *or n stable fixed points P*_1_ (*WTA procedure*) *together with one stable point P*_0_  *for b* < 0. *Other* 2^*n*^ − *n* − 2 *fixed points P*_*k*_, *k* = 2, …, *n* − 1, *are unstable points* (*saddles*) *for any values of the parameters*. *The point P*_*n*_ *is stable if n* ≥ 2 *and*

18 $$b > \mathrm{0,}\quad (c+\gamma )f^{\prime} \mathrm{(0)}+bg^{\prime} \mathrm{(0)} > \mathrm{0,}\quad \alpha (c+\gamma )f^{\prime} \mathrm{(0)} > 0.$$

*The point P*_1_ *is stable if*

19 $$b < \mathrm{0,}\quad (c+\gamma )f^{\prime} \mathrm{(0)}+nbg^{\prime} \mathrm{(0)} > \mathrm{0,}\quad \alpha (c+\gamma )f^{\prime} \mathrm{(0)} > 0.$$

Proposition 2 is proved in Appendix. For functions (8)–(10) with *f*′(0) = *ν* and *g*′(0) = 1 the conditions of stability for the point *P*_1_ (19) become simpler

$$-\,\frac{(c+\gamma )\nu }{n} < b < 0.$$

If *b* < 0 and the number of POs *n* is large enough

$$n\ge -\frac{(c+\gamma )\nu }{b},$$

then the system does not have any stable point *P*_1_.

The system has the Andronov-Hopf (AH) bifurcation at the point *P*_*k*_ when *k*(*c* + *γ*)*f*′(0) + *nb* = 0 and it has the pitchfork (PF) bifurcation at the point *P*_*k*_ when *α*(*c* + *γ*)*f*′(0) = 0. The AH bifurcation implies the existence of stable limit cycles around the former fixed points *P*_0_, *P*_1_, and *P*_*n*_, in other cases the limit cycles are of the saddle type. The PF bifurcation changes the dimension of the stable and unstable invariant manifolds for *P*_1_, …, *P*_*n*−1_, but it can not make these points stable.

System (16) of identical oscillators has the invariant manifold

$${\phi }_{i}=\phi ,\quad {a}_{i}=a,\quad i=1,\ldots ,n,$$

for arbitrary values of other parameters. This manifold corresponds to full synchronization. The dynamics on this manifold are described by 3D system (16) for *n* = 1. This system has two fixed points (0, *ω*, *c* + *γ*) and (*π*, *ω*, *c*) that correspond to inphase and antiphase synchronization of the CO with the PO. The first point is stable if (*c* + *γ*)*f*′(0) > −*bg*′(0), *α*(*c* + *γ*)*f*′(0) > 0. These conditions can be satisfied for both positive and negative values of *b*. The second point is stable in two directions when *b* < 0 and neutral in the third direction (one eigenvalue of the point is zero).

According to the permutation symmetry S_*n*_ system (16) has also the invariant manifolds

$${\phi }_{i}={\phi }_{j},\quad {a}_{i}={a}_{j},\quad i\ne j,$$

that correspond to the regime when two oscillators (*i*-th and *j*-th POs) form a synchronous cluster. In a similar way the system can have a cluster with *k* synchronous POs for any *k* = 2, …, *n*.

#### **Remark 1.**

The results presented in Proposition 2 correlate with our previous results^60^ about a star-like phase oscillator model with constant amplitudes of oscillations *a*_*i*_(*t*) = *a* = const. It has been shown that in this model different stable regimes Φ_*k*_ coexist for different *k* = 1, … *n*. The additional condition *f*′(*π*) = 0 insures that only three types of stable regimes are possible: global synchronization Φ_*n*_, WTA regime Φ_1_, and no-winner regime Φ_0_ (all other points Φ_*k*_ are saddles).

### Non–identical peripheral oscillators. Stationary solution

Equations for connection strengths (14) are linear non-homogeneous differential equations that have the solutions

$${a}_{i}(t)=c+({a}_{i}({t}_{0})-c){e}^{-\beta (t-{t}_{0})}+\beta \gamma {e}^{-\beta t}\,{\int }_{{t}_{0}}^{t}\,{e}^{\beta \tau }h({\phi }_{i}(\tau ))d\tau .$$

The assumption 0 ≤ *h*(*x*) ≤ 1 implies that

$$c+({a}_{i}({t}_{0})-c){e}^{-\beta (t-{t}_{0})}\le {a}_{i}(t)\le c+\gamma +({a}_{i}({t}_{0})-c-\gamma ){e}^{-\beta (t-{t}_{0})}.$$

The following proposition presents the boundaries for amplitude variables.

#### **Proposition 3.**

*For any ε* > 0 *there is a large enough moment of time t*_1_ *such that for any t* > *t*_1_

20 $$c-\varepsilon \le {a}_{i}(t)\le c+\gamma +\varepsilon ,\quad i=1,\ldots ,n.$$

*This inequality is valid for any initial conditions and parameter values*.

Let us start from the symmetric case when all natural frequencies are equal, *ω*_*i*_ = *ω*. In this case the system has *n* different symmetric fixed points *P*_1_ (see the previous subsection) which are stable for some parameter values (Proposition 2). To distinguish between these points, the following notation is helpful:

$$\begin{array}{ccc}{P}_{1,l} & = & ({\phi }_{1},\ldots ,{\phi }_{n},{\omega }_{0},{a}_{1},\ldots ,{a}_{n})\\  & = & (\pi ,\ldots ,\pi ,\mathop{\underbrace{0}}\limits_{l},\pi ,\ldots \pi ,\omega ,c,\ldots c,\mathop{\underbrace{c+\gamma }}\limits_{l},c,\ldots c),\quad l=1,\ldots ,n.\end{array}$$

The perturbation of natural frequencies leads to the displacement of the points *P*_1,*l*_. It also breaks the S_*n*_ symmetry of these points (the coordinates of the points are non-symmetric in the general case), but it cannot change the stability of the points because they are hyperbolic. Denote *Q*_*l*_ a perturbed location of the point *P*_1,*l*_ when *ω*_*l*_ = *ω* + Δ_*l*_, *l* = 1, …, *n*, Δ_*l*_ are relatively small: |Δ_*l*_| ≤ |*b*|. The point *Q*_*l*_ describes the WTA case when the *l*-th PO is the winner.

The following statement describes the stability of different WTA points *Q*_*l*_ in the case when the functions *f*, *g*, *h* are specified as (8)–(10) with large *ν* and *μ* < *π*/2.

#### **Proposition 4.**

*System* (12)–(14) *with functions* (8)–(10), *a large enough value of the parameter ν of the function f and small perturbation of the natural frequencies ω*_*i*_ = *ω* + Δ_*i*_, |Δ_*i*_| < |*b*|, *has n fixed points Q*_*l*_ *that correspond to the synchronization of the l*-*th PO with the CO*. *In this case ω*_0_ ≈ *ω*_*l*_, *that is the CO obtains the frequency near the frequency of the* “*winning*” *PO*. *Moreover*, *these oscillators work nearly inphase*, *while loser POs are radically incoherent with the CO*.

*The coordinates of Q*_*l*_ *can be approximately represented as*

21 $$({\bar{\phi }}_{1},\ldots ,{\bar{\phi }}_{l-1},\mathop{\underbrace{0}}\limits_{l},{\bar{\phi }}_{l+1},\ldots ,{\bar{\phi }}_{n},{\omega }_{l},c,\ldots ,c,\mathop{\underbrace{c+\gamma }}\limits_{l},c,\ldots c),$$

*where*

$${\bar{\phi }}_{i}\approx \pi -\arcsin (\frac{{\omega }_{i}-{\omega }_{l}}{b}),\quad i\ne l.$$

*The points Q*_*l*_ *are stable if*

22 $$b < 0,\quad (c+\gamma )\nu +nb > 0,\quad n\alpha (c+\gamma )\nu  > 0.$$

### Non–identical peripheral oscillators. Non-stationary solution

If the natural frequencies of POs are distributed in a large range, the CO has not enough strength to phase-lock all the POs. A stationary solution that describes WTA type of dynamics becomes impossible. Nevertheless, the main feature of the WTA regime when one PO works nearly inphase with the CO, while other POs are out of phase can survive even in this case. We consider here the solutions for which the variables *φ*_*l*_, *ω*_0_, *a*_1_, …, *a*_*n*_ are close to constants: the variable *φ*_*l*_ is near zero, the variable *ω*_0_ is near *ω*_*l*_, the variable *a*_*l*_ varies slightly below the level *c* + *γ*, and the variables *a*_*i*_, *i* ≠ *l*, vary slightly above the level *c*. These assumptions together with (8, 10) lead to the following approximate equations for the phases *φ*_*i*_:

23 $$\frac{d{\phi }_{i}}{dt}={{\rm{\Omega }}}_{i}-bg({\phi }_{i}),\quad i\ne l,$$

where Ω_*i*_ = *ω*_*i*_ − *ω*_*l*_. For a given *l* system (23) is a system on the torus ${{\mathbb{T}}}_{l}^{n-1}$. Note that the torus ${{\mathbb{T}}}_{l}^{n-1}$ is not an invariant set of system (12)–(14), but it is located very close to the true (*n* − 1)–dimensional invariant manifold ${ {\mathcal M} }_{l}^{n-1}$ (which has a more intricate structure). The manifold ${ {\mathcal M} }_{l}^{n-1}$ is stable in the transversal *n* + 2 dimensions and local dynamics inside this manifold are equivalent to the dynamics of system (23) on ${{\mathbb{T}}}_{l}^{n-1}$. Though each manifold ${ {\mathcal M} }_{l}^{n-1}$ is located approximately in the neighborhood of the hyperplane *φ*_*l*_ = 0, *ω*_0_ = *ω*_*l*_, *a*_*l*_ = *c* + *γ*, *a*_*i*_ = *c* (for *i* ≠ *l*), its geometry is nontrivial in the whole phase space ${{\mathbb{T}}}^{n}\times {\mathbb{R}}\times {{\mathbb{R}}}^{n}$ even in the case of identical oscillators. It becomes more and more complex with the complication of the natural frequencies distribution. This is why a strict mathematical proof of the existence of such manifold is not an easy task. We presume that such proof could be obtained using the normal hyperbolicity theory. Here we only note that the assumption about the existence of such manifolds ${ {\mathcal M} }_{l}^{n-1}$ with dynamics (23) is helpful for explaining all bifurcation transitions when the natural frequencies of oscillators are changed. The conclusions obtained by this reasoning are fully confirmed by numerical computations.

Without loss of generality let us order all natural frequencies of POs as

24 $${\omega }_{1}\ge {\omega }_{2}\ge \cdots \ge {\omega }_{n}.$$

**Figure 2:**
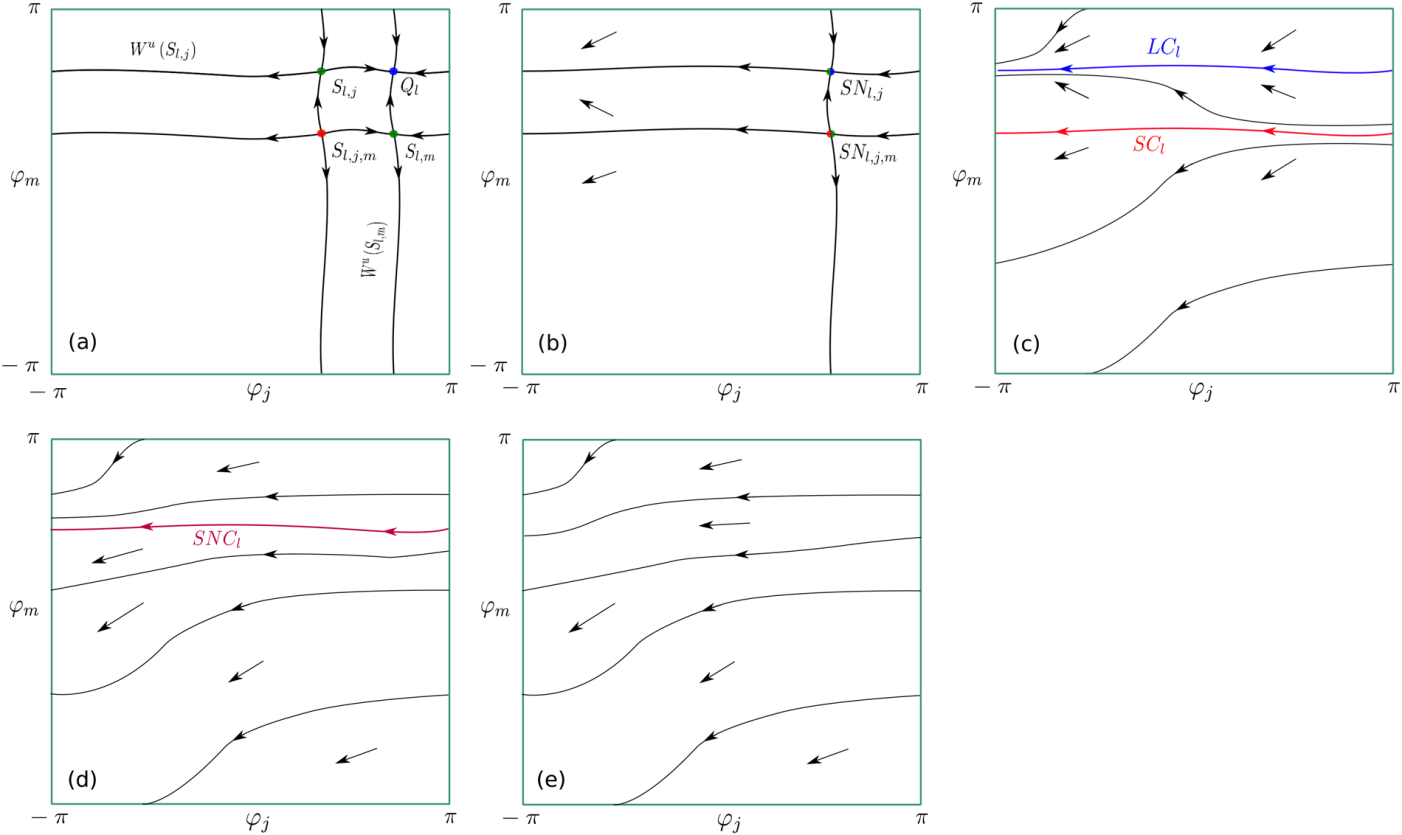
Bifurcation scheme. Schematic phase portraits on the 2–dimensional invariant toroidal manifold. Bifurcations occur inside the manifold which is stable in all transversal directions. Transitions of two simultaneous bifurcations are shown in (**a**–**c**). Stable and saddle cycles *LC*_*l*_ and *SC*_*l*_ appear after the corresponding bifurcations (**c**). Local saddle–node (fold) bifurcation of two cycles is a global bifurcation on the invariant toroidal manifold (SNIT bifurcation). It leads to the appearance of the 2–dimensional limit torus $L{T}_{l}^{2}$. The bifurcation transition inside the invariant toroidal manifold is shown in (**c**–**e**). *SNC*_1_ denotes a saddle node cycle. Stable (unstable) points and cycles are shown by blue (red) color, saddles are shown by green color.

**Figure 3:**
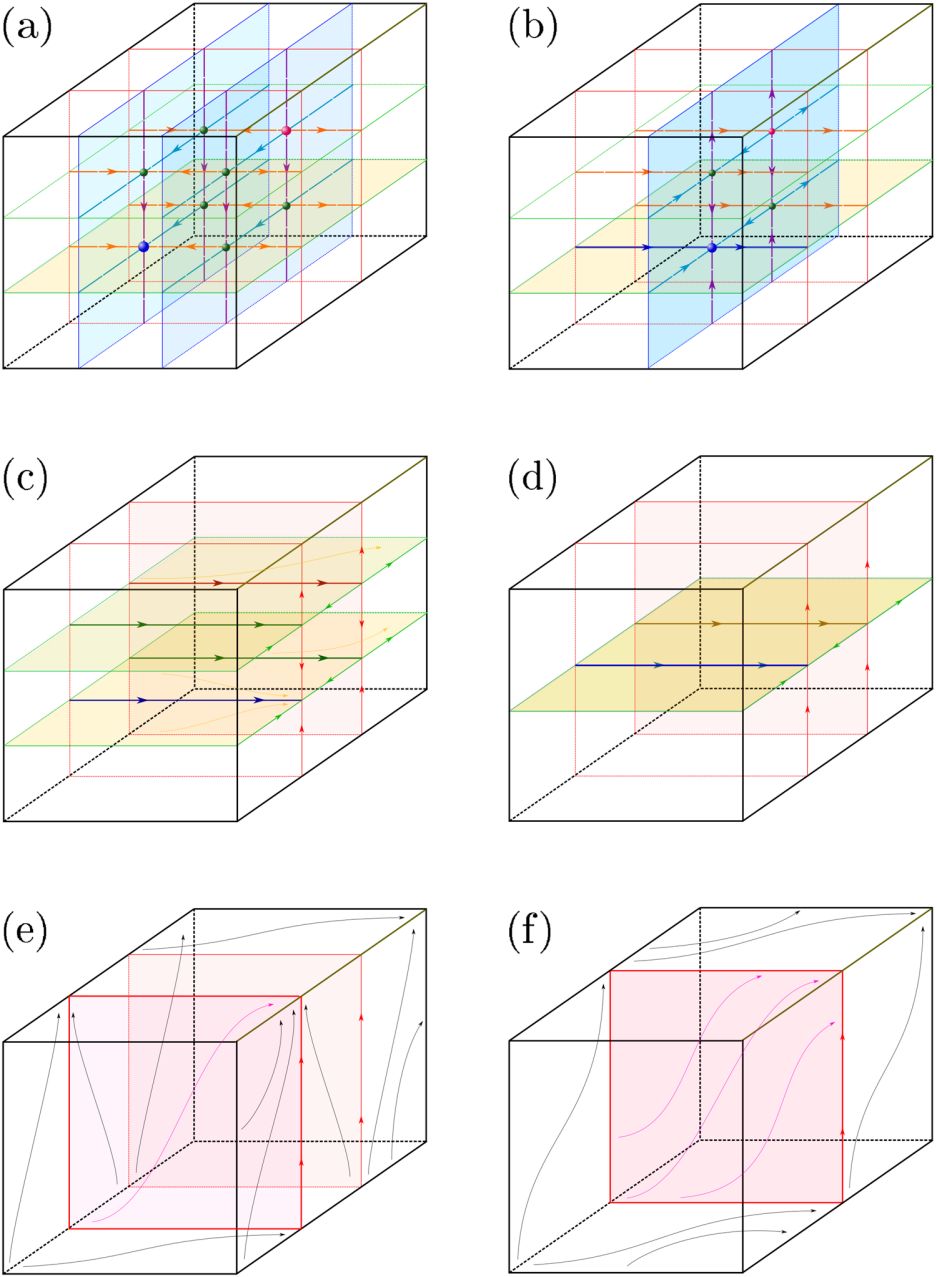
Scheme of SNIT bifurcation. The chain of bifurcations on the 3–dimensional torus ${{\mathbb{T}}}^{3}\ni ({\phi }_{i},{\phi }_{j},{\phi }_{m})$ is presented as a schematic phase diagrams. (**a**) Eight fixed points with 1D invariant manifolds (lines) and 2D invariant manifolds (planes). (**b**) Four simultaneous SNIC bifurcations (SN bifurcation of two 2D invariant manifolds on ${{\mathbb{T}}}^{3}$). (**c**) Appearance of four (stable, unstable and two saddle) limit cycles as a result of SNIC bifurcations. (**d**) Two simultaneous fold bifurcations of periodic orbits (SN bifurcation of two 2D invariant manifolds on ${{\mathbb{T}}}^{3}$). (**e**) Appearance of stable and unstable 2D limit surfaces (planes) as a result of disappearance of limit cycles. (**f**) SN bifurcation of stable and unstable limit surfaces on 3D torus (SNIT). Disappearance of the resolution surface leads to the permeability of the whole ${{\mathbb{T}}}^{3}$ for the trajectories.

We have shown (see Methods) that besides stable points *Q*_*l*_ there exist other saddle fixed points *S*_*l*,*j*_, *j* ≠ *l*, with only one different coordinate *φ*_*i*_. The local saddle-node bifurcation of these two points is also a global saddle-node on invariant cycle (SNIC) bifurcation along the mentioned coordinate *φ*_*i*_ (which belongs to the manifold ${ {\mathcal M} }_{l}^{n-1}$). Figure 2(a–c) shows disappearance of the stationary WTA regime *Q*_*l*_ and appearance of the non-stationary WTA regime *LC*_*l*_. Figure 3(a–c) illustrates the following proposition:

#### **Proposition 5.**

*SNIC bifurcations occur as a result of merging between the stable point Q*_*l*_ *and the saddle point S*_*l*_ *when*

$${\omega }_{l}-{\omega }_{n}=|b|,\quad but\quad {\omega }_{1}-{\omega }_{l} < |b|$$

*or when*

$${\omega }_{1}-{\omega }_{l}=|b|,\quad but\quad {\omega }_{l}-{\omega }_{n} < |b\mathrm{|}.$$

*A stable limit cycle LC*_*l*_ *appears along the 1*–*dimensional manifold of the saddle S*_*l*_ *with the unbounded coordinate φ*_*n*_ (*or φ*_1_ *in the second case*) *and bounded phase coordinates φ*_*i*_, *i* = 1, …, *n* − 1 (*or φ*_*i*_, *i* = 2, …, *n*, *respectively*). *If ω*_1_ − *ω*_*n*_ = |*b*|, *two SNIC bifurcations occur simultaneously with the points Q*_1_ *and Q*_*n*_ *giving birth to two stable limit cycles LC*_1_ *and LC*_*n*_.

The periodic trajectory *LC*_1_ rotates in the positive direction along *φ*_*n*_ with the average frequency ${\hat{\omega }}_{n}\in ({\omega }_{1}-{\omega }_{n}-|b|,\,{\omega }_{1}-{\omega }_{n}+|b|)$. Similarly, the average frequency for the cycle *LC*_*n*_ is ${\hat{\omega }}_{n}\in ({\omega }_{n}-{\omega }_{1}-$ $|b|,\,{\omega }_{n}-{\omega }_{1}+|b|)$and the rotation goes in the negative direction along *φ*_*n*_. The amplitudes of POs whose phases run on *LC*_1_ are also bounded (according to Proposition 3), *a*_1_ ≈ *c* + *γ*, *a*_*n*_ oscillates in a small neighborhood above the value *c* and all other amplitudes are approximately equal to *a*_*i*_ = *c*. Another SNIC bifurcation at the point *Q*_*l*_ occurs when the distance *ω*_*l*_ − *ω*_*n*_ (or the distance *ω*_1_ − *ω*_*l*_) is equal to |*b*| while the distance *ω*_1_ − *ω*_*l*_ (respectively, *ω*_*l*_ − *ω*_*n*_) is smaller than |*b*|. SNIC bifurcations happen with all the points *Q*_*s*_, *s* < *l*, (or with *Q*_*s*_, *s* > *l*, for *ω*_1_ − *ω*_*l*_ = |*b*|) before it happens with *Q*_*l*_ because *ω*_*s*_ − *ω*_*n*_ ≥ *ω*_*l*_ − *ω*_*n*_ = |*b*|.

Besides limit cycles, the non-stationary WTA regime can be associated with more complex solutions if the number of POs *n* ≥ 3 and there are several (more then one) POs for which the variables *φ*_*i*_ (*i* ≠ *l*) are not bounded but constantly run in the positive or negative direction in phase space. The dynamics on the high-dimensional toroidal manifold ${ {\mathcal M} }_{l}^{n-1}$, *n* ≥ 3, is adequately described by system (23). More complex WTA regimes (with some number of PO phases running relative to the phase of the CO) appear as a generalization of the SNIC bifurcation. We call this bifurcation *the Saddle*-*Node* (*fold*) *bifurcation on Invariant Torus* (SNIT). Similarly to the SNIC bifurcation of two points on the 1D torus (circle) ${{\mathbb{T}}}^{1}$, the SNIT bifurcation is a saddle-node (fold) bifurcation of the stable and saddle 1D cycles on the 2D torus ${{\mathbb{T}}}^{2}$ (which leads to full transitivity of the torus), or the bifurcation of stable and saddle 2D tori in 3D phase space ${{\mathbb{T}}}^{3}$, or (in the general case) the bifurcation of the stable and saddle (*m* − 1)-dimensional tori on *m*-dimensional torus ${{\mathbb{T}}}^{m}.$ These bifurcations are described by the following preposition (see Methods for more details):

#### **Proposition 6.**

*As a result of the SNIT bifurcation*, *a pair of the stable* $L{T}_{l}^{m-1}$ *and saddle* $S{T}_{l}^{m-1}$ *m*–*dimensional tori appears on a stable m*–*dimensional torus* $L{T}_{l}^{m}$. *This SNIT bifurcation can occur for any number m* = 2, …, *n* − 1, *when different m* − 1 *values of* |(*ω*_*l*_ − *ω*_*i*_)/*b*| − 1, *i* ≠ *l*, *are positive*, *one value is zero*, *and n* − *m* − 1 *values are negative*.

The dynamics on the invariant torus $L{T}_{l}^{m}$ can be very complex starting from the dimension *m* = 2. A lot of useful information on this subject can be found in the classical works^68,69,74^ (especially for the case ${{\mathbb{T}}}^{2}$). SNIT transitions of *LC*_*l*_ and *SL*_*l*_ on ${{\mathbb{T}}}^{2}$ are schematically presented in Figs 2(c–e) and 3(c–e) (two simultaneous SNIT bifurcations on two parallel 2D tori), The SNIT bifurcation of 2D tori $L{T}_{l}^{2}$ and $S{T}_{l}^{2}$ on ${{\mathbb{T}}}^{3}$ is presented in Fig. 3(e,f). In the two dimensional case the SNIT bifurcation occurs in the literature under alternative names, like *a boundary of partial mode*-*locking* (if one takes a Poincaré map, then it is the boundary of an Arnold tongue) or *a saddle*-*node periodic orbit with the global reinjection*. The principal parts of the structure of a similar bifurcation but without reinjection for *m* = 3 were described in detail in^71,73^. As far as we know, there is no mentioning of SNIT bifurcations in the literature for the cases *m* ≥ 4 and for *m* = 3 with the global reinjection. It is presented here for the firs time.

**Figure 4:**
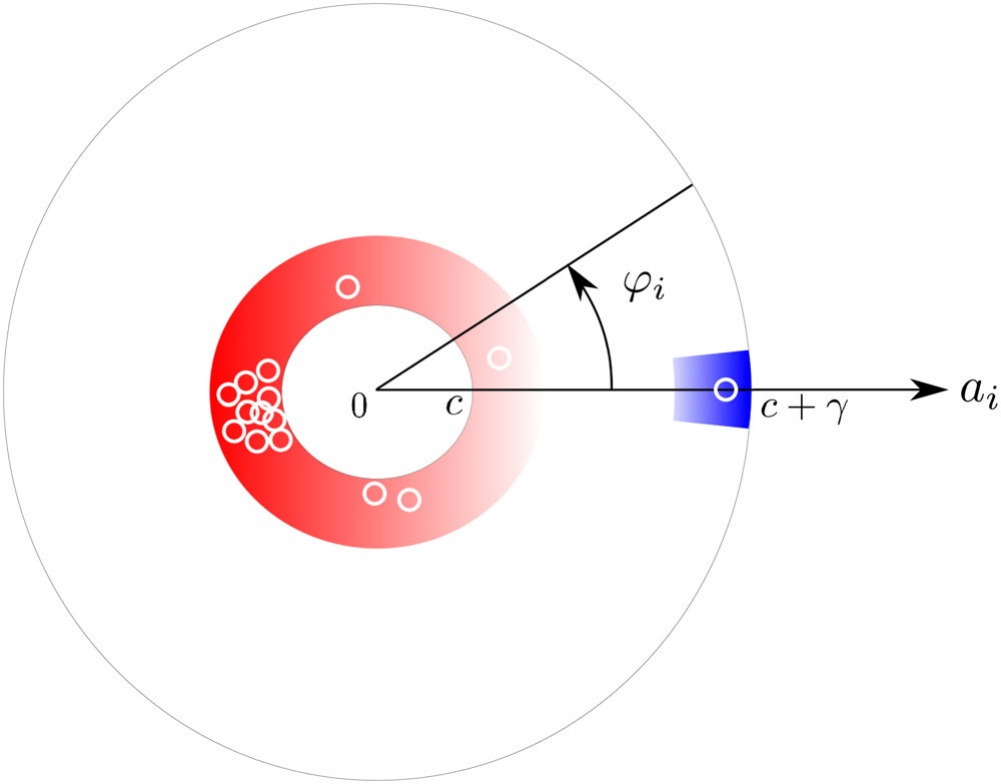
Distribution of phases and amplitudes. Schematic diagram of distribution of oscillators with amplitudes *a*_*i*_ and phase differences *φ*_*i*_. Blue colour indicates the location of the winner, red color indicates the location of the losers. Darker colour corresponds to higher probability of location of oscillators in the marked area. Small open circles with white boarders correspond to separate POs.

A diagram of POs distribution on the plane (*a*_*i*_,*φ*_*i*_) is schematically shown in Fig. 4. All amplitudes are located inside the area *a*_*i*_ ∈ [*c*, *c* + *γ*], the winning PO moves somewhere near the point (*c* + *γ*, 0) (the area is shown in blue color). Loser POs are distributed in the red area. Some of them form a cluster near the point (*c*,*π*), while others run around in the red ring area close to the circle *a*_*i*_ = *c*.

#### **Remark 2.**

The results obtained in this subsection can be generalized for the functions *f*, *g*, *h* more general or slightly different from those defined in (8)–(10). For example, similar results can be obtained for an arbitrary periodic function *g*(*x*) (not necessary odd) that satisfies the second and third conditions in (6), *g*(*x*) > 0 for $x\in \mathrm{(0},\pi )$, *g*(*x*) < 0 for *x* ∈ (−*π*, 0), max*g*(*x*) = −min *g*(*x*) = 1. In this case bifurcation conditions depend on the values of *x*_max_ and *x*_min_ of the function *g*(*x*) in addition to the values of *ω*_*i*_ and *b*. The results similar to those above can also be obtained without assuming oddness of the function *f*(*x*). The only critical assumptions are high values for the slope *f*′(0) and zero slope *f*′(*π*). Evenness of the function *h*(*x*) is also not necessary, but other conditions (7) must be fulfilled.

### No-winner solutions

According to Proposition 2, system (12)–(14) with identical POs has not only WTA fixed points *P*_1_ but also the fixed point *P*_0_ = (*φ*_1_, …, *φ*_*n*_, *ω*_0_, *a*_1_, …, *a*_*n*_) = (*π*, …, *π*, *ω*, *c*, … *c*) that corresponds to a regime, where all POs are in antiphase to the CO (that is there are no winners). In contrast to the points *P*_1_ (which can be asymptotically stable when *b* < 0) the point *P*_0_ is stable in 2*n* directions and it is neutral along the last direction (see Lemma 1 in Methods). The neutral direction of *P*_0_ is along the straight line that can be described as one parametric set of initial conditions for the natural frequency of the CO ${\tilde{\omega }}_{0}={\omega }_{0}\mathrm{(0)}$ as

$$L({\mathop{\omega }\limits^{ \sim }}_{0})=\{({\phi }_{1},\ldots ,{\phi }_{n},{\omega }_{0},{a}_{1},\ldots ,{a}_{n})\in {{\mathbb{T}}}^{n}\times {{\mathbb{R}}\times {\mathbb{R}}}^{n}:{\phi }_{i}=\mathop{\phi }\limits^{ \sim },\,{\omega }_{0}={\mathop{\omega }\limits^{ \sim }}_{0},\,{a}_{i}=c,\,i=1,\ldots n\},$$

where

$$\mathop{\phi }\limits^{ \sim }=\pi +{g}^{-1}(\frac{\omega -{\mathop{\omega }\limits^{ \sim }}_{0}}{b}),\quad {g}^{-1}\,{\rm{i}}{\rm{s}}\,{\rm{t}}{\rm{h}}{\rm{e}}\,{\rm{i}}{\rm{n}}{\rm{v}}{\rm{e}}{\rm{r}}{\rm{s}}{\rm{e}}\,{\rm{f}}{\rm{u}}{\rm{n}}{\rm{c}}{\rm{t}}{\rm{i}}{\rm{o}}{\rm{n}}\,{\rm{t}}{\rm{o}}\,g.$$

According to Proposition 1, the fixed point *P*_0_ is isolated, there are no other fixed points in a small neighborhood of this point. However, considering specific functions (8)–(10) for the system we have: *dφ*_*i*_/*dt* ≈ 0, *dω*_0_/*dt* ≈ 0, *dh*/*dt* = 0, *i* = 1, … *n*, when the phase point belongs to $L({\tilde{\omega }}_{0})$ in a small neighborhood of *P*_0_. As a result, computer simulations of the system “perceive” the points of $L({\tilde{\omega }}_{0})$ in the neighborhood of *P*_0_ as fixed points that are neutral along *L* and stable in other directions.

Consider the system with functions (8)–(10) and perturbed frequencies *ω*_*i*_ = *ω* + Δ_*i*_, *i* = 1, …, *n*. The perturbed point *P*_0_(*ω*_1_, …, *ω*_*n*_) moves smoothly in phase space from the location *P*_0_(*ω*, …, *ω*) = *P*_0_ with the perturbation of natural frequencies of POs. The perturbation of the frequencies does not change amplitude coordinates *a*_*i*_ = *c* for a wide region of natural frequencies. Our computer simulations show that the perturbed point *P*_0_(*ω*_1_, …, *ω*_*n*_) remains stable in 2*n* directions and neutral in a single direction. The neutral line *L* of the point *P*_0_(*ω*_1_, …, *ω*_*n*_) moves with this point and rotates in phase space depending on the frequency distribution. The coordinate *ω*_0_ of the perturbed point is approximately equal to (*ω*_max_ + *ω*_min_)/2, i.e. only the fastest and slowest POs determine the average frequency of the CO in the no–winner situation. There are a few scenarios of disappearance of the point *P*_0_(*ω*_1_, …, *ω*_*n*_) which are based on a saddle–node bifurcation of this point with another saddle point (or points) depending on the system dimension and frequency distribution. In any case, the region where the mentioned fixed point exists belongs to the region which is bounded by the condition *ω*_max_ − *ω*_min_ < 2|*b*|. According to (26), the point *P*_0_ has *n* eigenvalues ${\lambda }_{2}({P}_{0})=\cdots ={\lambda }_{n+1}({P}_{0})=b$. A perturbation of *ω*_*i*_ leads to the (nonuniform) decrease of the corresponding eigenvalues |*λ*_*i*_(*P*_0_(*ω*_1_, …, *ω*_*n*_))|, *i* = 2, …, *n* + 1, which also indicates a restriction put on the attraction basin of *P*_0_(*ω*_1_, …, *ω*_*n*_).

### Examples of dynamics

In this subsection we give some examples of different types of dynamics of system (12)–(14), pointing out the bifurcations that split these examples. The following examples will be considered:

1. Identical natural frequencies of POs: *ω*_*i*_ = *ω*, *i* = 1, …, *n*. According to Proposition 1, system (12)–(14) has *n S*_*N*_–symmetrical stable fixed points *P*_1,*i*_ with coordinates (17).
2. Non-identical natural frequencies of POs that satisfy the inequalities
  $$|\frac{{\omega }_{i}-{\omega }_{j}}{b}| < 1,\quad i,j=1,\ldots ,n\,\Longleftrightarrow \,\mathop{max}\limits_{i\in \{1,\ldots n\}}{\omega }_{i}-\mathop{min}\limits_{i\in \{1,\ldots n\}}{\omega }_{i} < |b|.$$
  In this case system (12)–(14) has *n* non-symmetrical stable fixed points *Q*_*l*_, *l* = 1, …, *n*, with coordinates
  $${\phi }_{l}\approx \mathrm{0,}\quad {a}_{l}\approx c+\gamma ,\quad {\omega }_{0}\approx {\omega }_{l},\quad |{\phi }_{i}-\pi | < \frac{\pi }{2},\quad {a}_{i}\approx c,\quad i\ne l.$$
3. The natural frequencies of POs satisfy the equality
  $$\mathop{{\rm{\max }}}\limits_{i\in \{\mathrm{1,}\ldots n\}}{\omega }_{i}-\mathop{{\rm{\min }}}\limits_{i\in \{\mathrm{1,}\ldots n\}}{\omega }_{i}=|b\mathrm{|}.$$
  In this case two simultaneous saddle-node bifurcations occur with two stable points ${Q}_{{i}_{{\rm{\max }}}}$, ${Q}_{{i}_{{\rm{\min }}}}$ with corresponding coordinates *ω*_*max*_ = max_*i*_ *ω*_*i*_, *ω*_*min*_ = min_*i*_ *ω*_*i*_ and saddles ${S}_{{i}_{{\rm{\max }}}},$ ${S}_{{i}_{{\rm{\min }}}}$. In this case the saddle–nodes also have the coordinates ${\phi }_{{i}_{{\rm{\max }}}}=\pi \mathrm{/2}$, ${\phi }_{{i}_{{\rm{\min }}}}=\pi \mathrm{/2}$, respectively. It is possible that more than two simultaneous saddle–node bifurcations take place if more than one natural frequency reaches the same maximal (minimal) value max_*i*_ *ω*_*i*_ (min_*i*_ *ω*_*i*_) (this bifurcation transition is schematically represented in Figs 2(a–c) and 3(a–c)).
4. At least one pair of the natural frequencies satisfies the inequality
  $$|\frac{{\omega }_{i}-{\omega }_{j}}{b}| > 1\,\Longleftrightarrow \,\mathop{max}\limits_{i\in \{1,\ldots n\}}{\omega }_{i}-\mathop{min}\limits_{i\in \{1,\ldots n\}}{\omega }_{i} > |b|.$$
  This implies that the point ${Q}_{{i}_{{\rm{\max }}}}$ $({\rm{or}}\,{Q}_{{i}_{{\rm{\min }}}})$ disappears after the saddle–node bifurcation and a new stable limit cycle appears. The local saddle–node bifurcation is a global SNIC bifurcation by which a limit cycle appears from the 1–dimensional unstable invariant manifold of the saddle ${S}_{{i}_{{\rm{\max }}}}$ $({\rm{or}}\,{S}_{{i}_{{\rm{\min }}}})$. The appearance of the limit cycle is possible because the mentioned 1–dimensional invariant manifold of ${S}_{{i}_{{\rm{\max }}}}$ reaches the appropriate stable point ${Q}_{{i}_{{\rm{\max }}}}$ along the coordinate ${\phi }_{{i}_{{\rm{\max }}}}$ that is closed on the torus. Stable fixed points *Q*_2_, …, *Q*_*n*−1_ and two stable limit cycles *LC*_1_, *LC*_*n*_ co-exist after the first SNIC bifurcation. The system can have up to *n* − 1 limit cycles *LC*_*i*_.
5. The first SNIT bifurcation and the appearance of the first 2–dimensional limit torus $L{T}_{1}^{2}$ (or $L{T}_{n}^{2}$) occur when |(*ω*_1_ − *ω*_*n*−1_)/*b*| = 1 (or when |(*ω*_2_ − *ω*_*n*_)/*b*| = 1). In this case a stable limit torus $L{T}_{1}^{2}$ and stable limit cycle *LC*_*n*−1_ appear simultaneously and exist for |(*ω*_1_ − *ω*_*n*−1_)/*b*| > 1 (or the limit torus $L{T}_{n}^{2}$ and stable limit cycle *LC*_2_ appear simultaneously).
6. Further increase of frequency differences leads to the appearance of new pairs of SNIC and SNIT bifurcations according to Proposition 6 and for increasing dimensions of new attractors by one (${Q}_{i}\,\mapsto \,L{C}_{i}$, $L{C}_{i}\,\mapsto \,L{T}_{i}^{2}$, $L{C}_{i}^{m-1}\,\mapsto \,L{T}_{i}^{m}$, *i* = 3, … *n* − 1). The sequences of SNIC and SNIT bifurcations that lead to the mentioned transitions (${Q}_{i}\,\mapsto \,L{T}_{i}^{n-1}$) are schematically represented on the 2-dimensional torus in Fig. 2 and on the 3-dimensional torus in Fig. 3.
7. In the most general case the system has limit tori $L{T}_{i}^{m}$, *m* = 0, …, *n* − 1, where $L{T}_{i}^{1}=L{C}_{i}$ is a limit cycle and $L{T}_{i}^{0}$ is a fixed point. There is multi-stability of *n* attractors of different types that correspond to the solutions of the WTA type. In each case the frequency of the CO adapts to the frequency of the winning PO.

According to Proposition 3 the amplitude of each PO *a*_*i*_(*t*) belongs to the interval [*c*, *c* + *γ*]. The amplitude of the winning PO *a*_*l*_ is close to the value *c* + *γ* or it can also oscillate below but very close to this value. If |(*ω*_*l*_ − *ω*_*i*_)/*b*| > 1 the amplitude *a*_*i*_ of a loser PO oscillates above the value *c* so that ${{\rm{\max }}}_{t > {t}_{1}}{a}_{i}(t)$ is significantly lower than *c* + *γ*. The value of max_*i*_ *a*_*i*_ depends on the parameters *β* and |*ω*_*i*_ − *ω*_*l*_/*b*|. Our numerical experiments show that max_*i*_ *a*_*i*_ grows approximately as $\beta /(\beta +|{\omega }_{i}-{\omega }_{l}/b|+{\tilde{\omega }}_{il})$, where ${\tilde{\omega }}_{il}$ is a constant.

#### **Example 1.**

**Figure 5:**
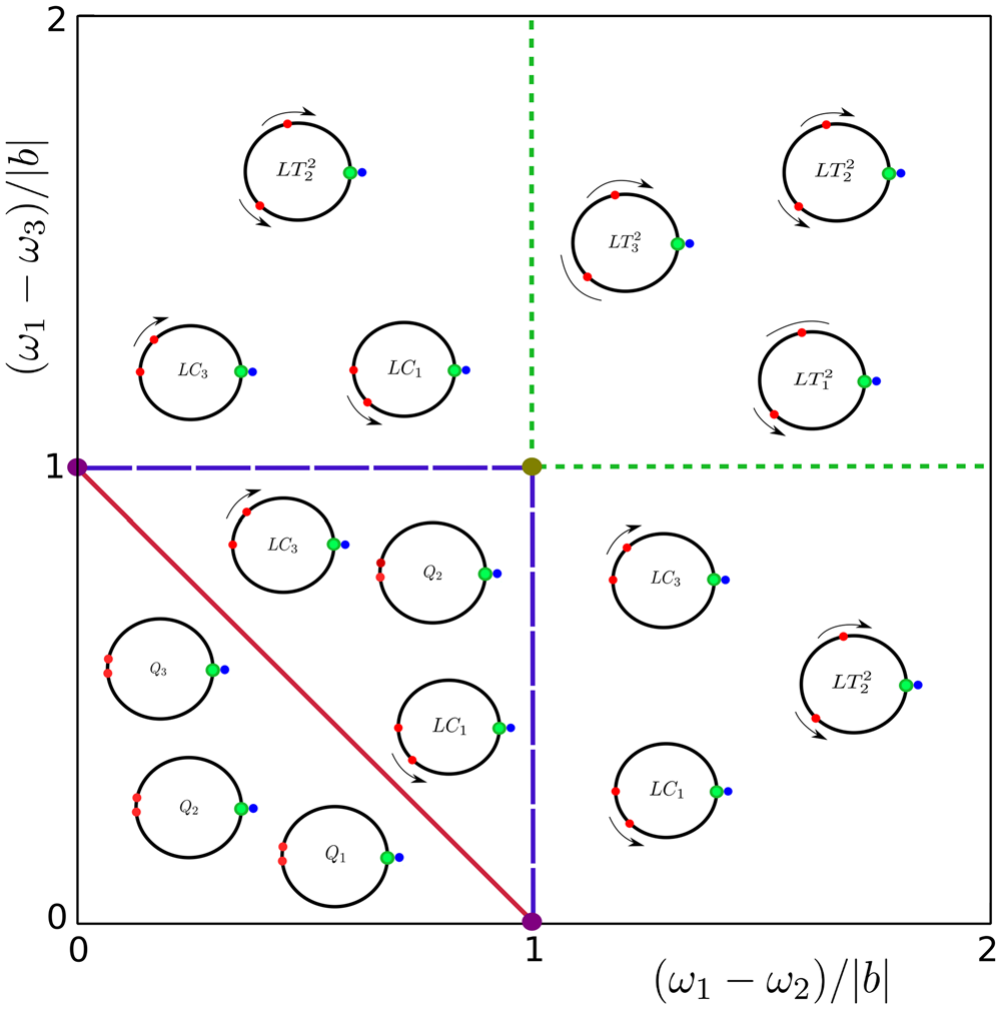
Bifurcations for 3 POs. Bifurcation diagram in the case *n* = 3 on bifurcation plane ((*ω*_1_ − *ω*_2_)/|*b*|, (*ω*_2_ − *ω*_3_)/|*b*|). Dark red line is the boundary for a SNIC bifurcation, dark blue and dark green lines show the boundaries for SNIT bifurcations. All WTA regimes in different bifurcation areas are represented as distributions on the circle of the phases of the CO (green), of the winning PO (blue), and of loser POs (red).

**Figure 6:**
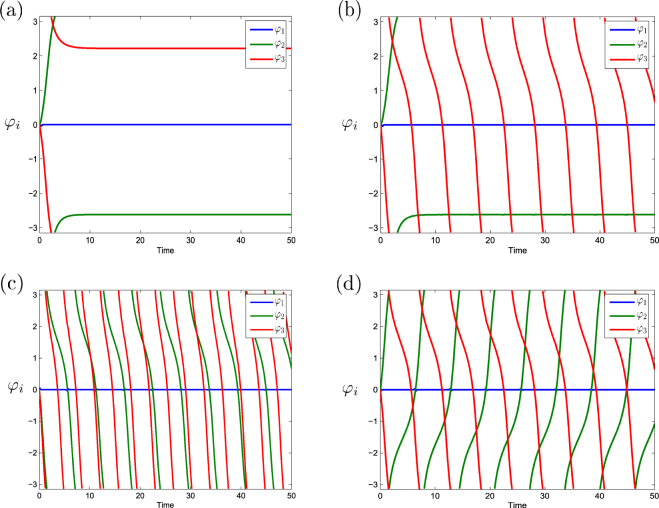
Time series of phase differences *φ*_*i*_, *i* = 1, 2, 3. The graphs show WTA solutions (the first PO is the winner) for four qualitatively different types of dynamics (illustrated in Fig. 5): (**a**) *Q*_2_, all POs are phase-locked by the CO, (**b**) *LC*_3_, two POs are phase-locked by the CO, the phase difference for the third PO runs in negative direction, (**c**) $L{T}_{3}^{2}$, one PO is the winner, the phase differences of the other two POs run in negative direction, (**d**), $L{T}_{2}^{2}$, one PO is the winner, the phase differences of the other two POs run in opposite directions. Initial values in (**a**–**d**) are *φ*_*i*_(0) = 0, *i* = 1, 2, 3, *ω*_0_(0) = 5, *a*_1_(0) = 13, *a*_2_(0) = 9, *a*_3_(0) = 1. The natural frequencies of POs are *ω*_1_ = 5 in (**a**–**d**), *ω*_2_ = 5.5, *ω*_3_ = 4.2 in (**a**), *ω*_2_ = 5.5, *ω*_3_ = 3.5 in (**b**), *ω*_2_ = 3.5, *ω*_3_ = 3 in (**c**), and *ω*_2_ = 6.4, *ω*_3_ = 3.5 in (**d**).

Consider system (12)–(14) with 3 POs. All possible types of oscillator dynamics depending on the distribution of the natural frequencies of POs on the bifurcation plane ((*ω*_1_ − *ω*_2_)/|*b*|, (*ω*_1_ − *ω*_3_)/|*b*|) are shown in Fig. 5. Bifurcation lines on this diagram correspond to the SNIC and SNIT bifurcations on the invariant manifold ${ {\mathcal M} }_{l}^{2}$ (see expression (23) and Propositions 5, 6). These bifurcations are schematically shown in Fig. 2. The graphs of phase differences *φ*_*i*_, *i* = 1, 2, 3, as functions of time are shown in Fig. 6. The parameters used in creation of this figure are *α* = 1, *β* = 0.05, *γ* = 10, *μ* = 1, *ν* = 20, *σ* = 100, *c* = 2, *b* = −1. Initial values are *φ*_*i*_(0) = 0, *i* = 1, 2, 3, *ω*_0_(0) = 5, *a*_1_(0) = 13, *a*_2_(0) = 9, *a*_3_(0) = 1. The values of the natural frequencies of POs are given in the legend of Fig. 6. The figure shows that in the WTA regime the CO can phase lock 1, 2, or 3 POs. In the first case the phase differences of non-synchronized POs can move in the same direction (positive or negative) or they can move in opposite directions. In the second case the phase difference of one non-synchronized PO is nearly stable while the phase difference of the other non-synchronized PO moves in positive or negative direction. In the third case all phase differences are stable. In any case, in this example the amplitude of the winning PO tends to 12 and the amplitudes of loser POs are near 2.

#### **Example 2.**

**Figure 7:**
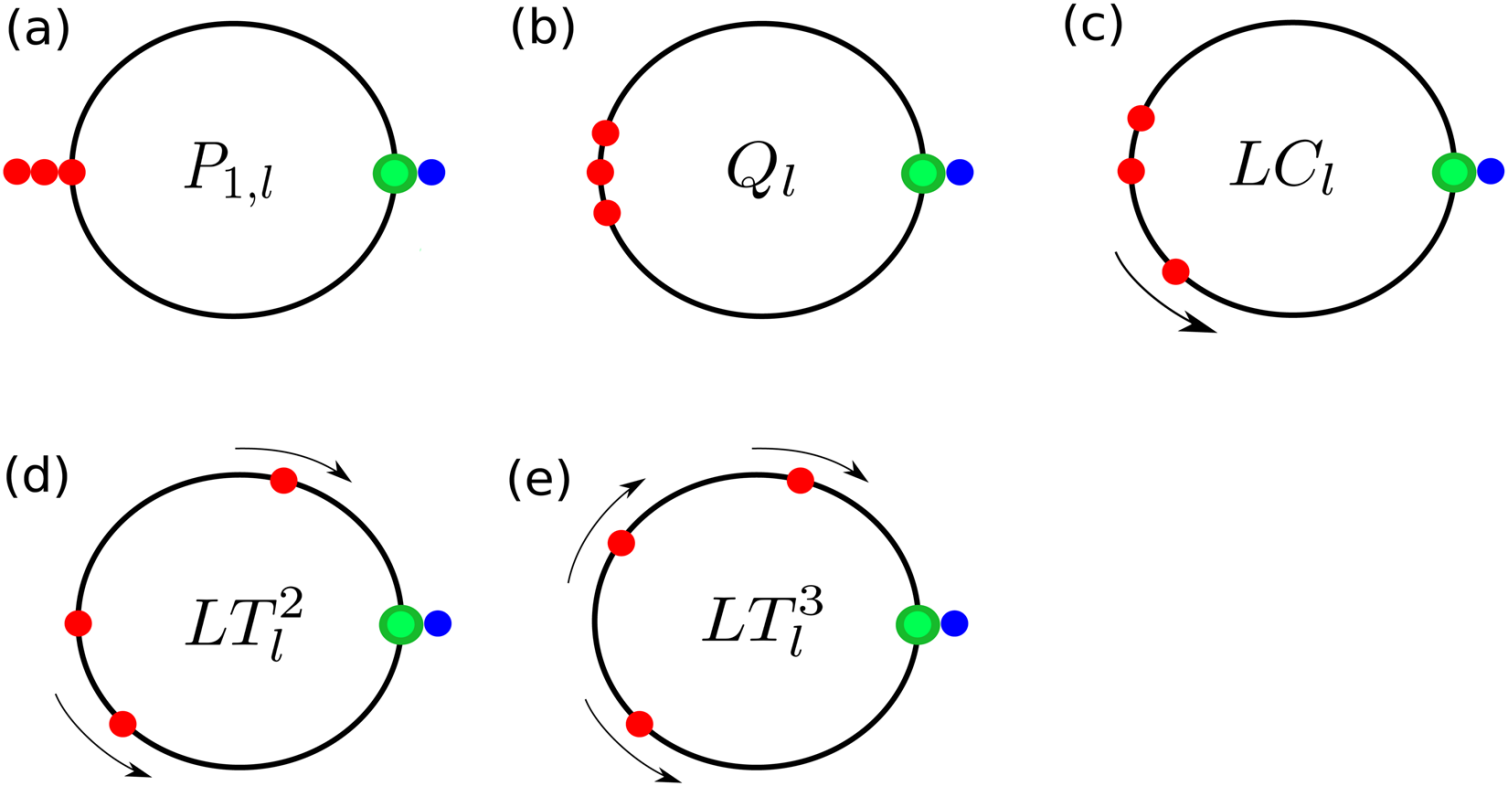
Distributions diagram for 4 POs. Distribution of the phases of the CO and four POs on the phase circle showing different WTA regimes. One PO synchronizes with the CO, while loser PO works close to the antiphase regime relative to the CO or rotates around the phase circle being incoherent with the CO. The colors are as in Fig. 5.

Figure 7 shows different types of WTA dynamics that can appear in the system with 4 POs. The corresponding types of attractors for system (12)–(14) with the natural frequencies (24) are also indicated: (a) *P*_1,*l*_ for ${\omega }_{1}=\cdots ={\omega }_{4}$; (b) *Q*_*l*_ for *ω*_1_ − *ω*_4_ < |*b*|; (c) *LC*_*l*_ for *ω*_1_ − *ω*_4_ > |*b*|, *ω*_1_ − *ω*_3_ < |*b*|, *ω*_2_ − *ω*_4_ < |*b*|; (d) $L{T}_{l}^{2}$ for *ω*_1_ − *ω*_3_ > |*b*|, *ω*_3_ − *ω*_4_ < |*b*| or *ω*_1_ − *ω*_2_ < |*b*|, *ω*_2_ − *ω*_4_ > |*b*|; (e) $L{T}_{l}^{3}$ for *ω*_*i*_ − *ω*_*i*+1_ > |*b*|, *i* = 1, 2, 3.

### Massive simulations

In this section we present different types of dynamics in terms of the frequencies with which they may appear in system (1)–(4) with functions (8)–(10). To check whether the WTA regime can be implemented by the system, we assign to *a*_1_ an initial value that is greater than initial values of the amplitudes of other POs. It is expected that in this case the first PO will have a greater chance to win the competition for synchronization with the CO. In simulations we set 4 ≤ *a*_1_(0) ≤ 12, while the initial values of other amplitudes are *a*_*i*_(0) = 2, *i* ≠ 1. During a simulation, the connection strengths are adapted according to equation (4). The values of the parameters in (4) are *c* = 2, *γ* = 10. This means that in the stationary case the amplitude of the winner will tend to the value *c* + *γ* = 12, while the other amplitudes will tend to *c* = 2. In the non-stationary case the amplitude of the winner varies in a narrow range around 12 and the amplitudes of the other POs vary around 2.

The difference in asymptotic dynamics of POs can be determined by checking whether the trajectory of a PO’s amplitude approaches the lower or higher boundary. Before checking this, some time should pass for the transient dynamics of the system to disappear. This time is set to be *T*_1_ = 80. The following 20 time units are used to determine the type of dynamics which are demonstrated by the system. Thus the whole run of the simulation takes *T*_2_ = 100 time units.

Two thresholds are introduced, *H*_*high*_ and *H*_*low*_., such that *c* < *H*_*low*_ < *H*_*high*_ < *c* + *γ*. In computations we set *H*_*high*_ = 10 and *H*_*low*_ = 3. If during the time interval (*T*_1_, *T*_2_) the trajectory of *a*_*i*_(*t*) lies above *H*_*high*_, then the *i*-th PO is identified as the winner.

All parameter values for different simulation examples are summarized in Table 1.

**Table 1:** Parameter values.

Parameters	Values				
	Ex. 3	Ex. 4	Ex. 5	Ex. 6	Ex. 7
The number of POs *n*	10	10	10	10	10
Initial phase of the CO *θ*_0_(0)	0	0	0	0	0
Initial phases of POs *θ*_*i*_(0)	0	0	0	0	0
				(−*π*/8, *π*/8)	
				(−*π*/4, *π*/4)	
				(−*π*/2, *π*/2)	
				(−*π*, *π*)	
Initial natural frequency of the CO *ω*_0_(0)	5	5	5	5	5
Natural frequencies of POs *ω*_*i*_	(4.9, 5.1)	(4.9, 5.1)	(4.25, 5.75)	(4.9, 5.1)	(4.9, 5.1)
Parameter *ν* of function *f*	20	5, 10, 15 20	5, 10, 15 20	20	20
Parameter *μ* of function *h*	1	1	1	1	1
Parameter *σ* of function *h*	100	100	100	100	100
Initial amplitude *a*_1_(0)	4, 6, 8, 10, 12	8	8	8	8
Initial amplitudes *a*_*i*_(0), *i* ≠ 1	2	2	2	2	2
Parameter *b* of equation (2)	−1	−1	−1	−1	−1
Parameter *α* of equation (3)	1	1	1	1	1
Parameter *c* of equation (4)	2	2	2	2	2
Parameter *γ* of equation (4)	10	10	10	10	10
Parameter *β* of equation (4)	0.05	0.05	0.05	0.05	0.05, 0.1, 0.15, 0.02
Time interval (*T*_1_, *T*_2_)	(80, 100)	(80, 100)	(80, 100)	(80, 100)	(80, 100)
Upper threshold *H*_*high*_	10	10	10	10	10
Lower threshold *H*_*low*_	3	3	3	3	3

Stochasticity is included in the simulations through initial values. First, the natural frequencies of POs are randomly distributed in the interval (4.9, 5.1) in Examples 3, 4, 6, 7 and in the interval (4.25, 5.75) in Example 4. Second, initial phases of POs are randomly distributed in one of the ranges (−*π*/8, *π*/8), (−*π*/4, *π*/4), (−*π*/2, *π*/2), (−*π*, *π*) in Example 6. In other examples all initial phases of the POs are 0. The initial value of the natural frequency of the CO is equal to 5 in all examples, which is the mean of the distribution of the natural frequencies of POs. The initial value of the phase of the CO is always 0.

Massive computational experiments show that the following three types of dynamics can be observed:

A. *a*_1_(*t*) > *H*_*high*_ for *T*_1_ < *t* < *T*_2_ and the first PO is the only oscillator for which this inequality is valid. This means that the first PO is the winner of the competition for the synchronization with the CO.
B. *a*_*i*_(*t*) > *H*_*high*_ for *T*_1_ < *t* < *T*_2_ and a single value of the index *i* ≠ 1. This means that the first PO lost the competition despite its initial advantage in influencing on the CO.
C. *a*_*i*_(*t*) < *H*_*low*_ for *T*_1_ < *t* < *T*_2_ and all *i* = 1, …, *n*. This is the case when there is no winner of the competition.

Note that due to Proposition 2 there cannot be more than one winner of the competition in the stationary case. Simulations show that the same situation is valid for a non-stationary case too. Another important fact is that no chaotic behavior has been observed in the system for the parameter values presented in Table 1.

It is reasonable to think that a WTA system demonstrates better performance if in most cases its dynamics correspond to situation (A), while the other cases occur only rarely (if at all). In particular, the probability of the (C)-type dynamics should be minimized, since in this case the result of the competition is indefinite.

**Figure 8:**
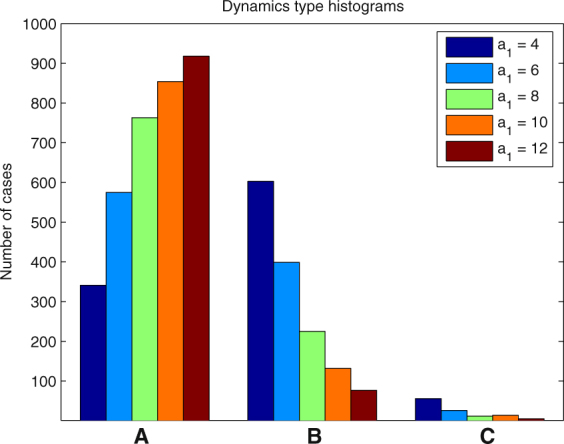
Histogram for Example 3. The probability that the first PO wins the competition increases when *a*_1_(0) increases.

**Figure 9:**
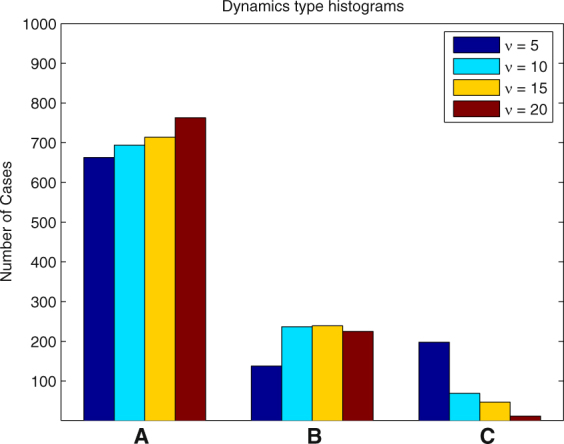
Histogram for Example 4. Greater steepness *ν* of the function *f* at 0 results in increasing probability of case (**A**) and decreasing probability of case (**C**).

**Figure 10:**
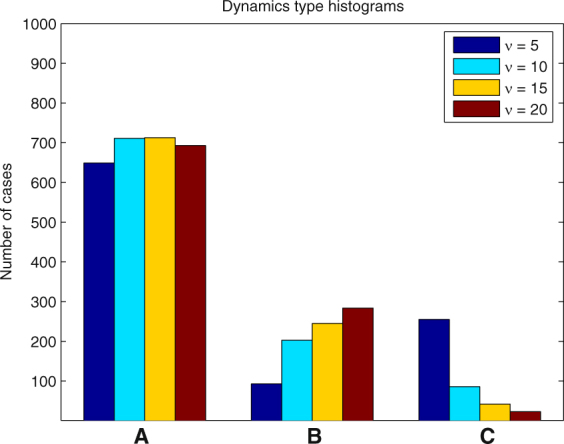
Histogram for Example 5. The WTA regime in the non-stationary case.

**Figure 11:**
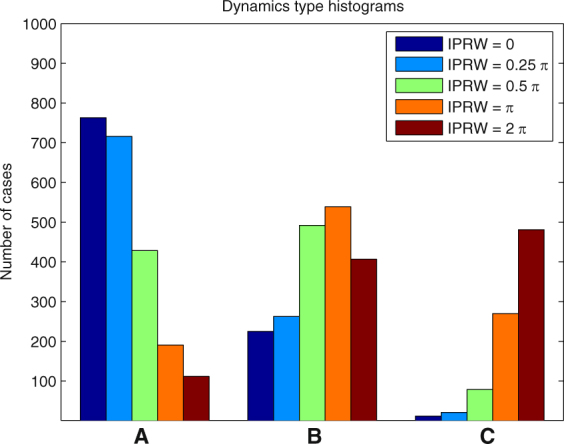
Histogram for Example 6. The performance of the WTA regime rapidly decreases when the initial phase range width (IPRW) increases.

**Figure 12:**
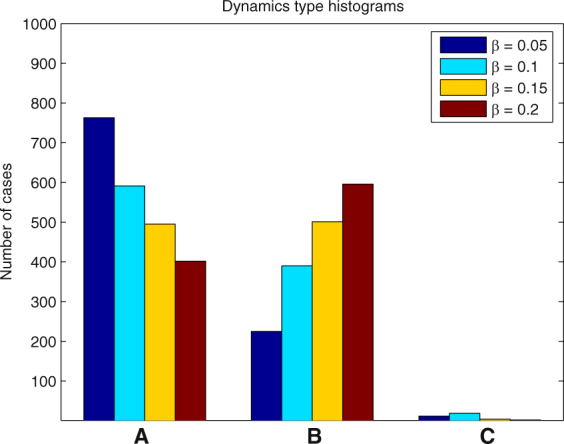
Histogram for Example 7. Slow adaptation of PO’s amplitudes is preferable for WTA performance.

In each of the following examples we ran 1000 simulations and determined the number of cases when one of the types of dynamics (A)-(C) took place. The results for different parameter values are presented in the form of histograms in Figs 8, 9, 10, 11 and 12.

#### **Example 3.**

In this example we vary the initial value of the amplitude of the first PO *a*_1_(0) consecutively assigning to it the values 4, 6, 8, 10, 12. It is expected that the higher the value of *a*_1_(0), the greater the chance that the first PO will win the competition. This expectation is confirmed by Fig. 8. The probability that the first PO wins the competition increases from 34.1% for *a*_1_(0) = 4 to 91.8% for *a*_1_(0) = 12. Respectively, the probability that another PO wins the competition decreases from 60.3% to 7.7%. The probability that case (C) will be the outcome of the competition is always low and decreases when *a*_1_(0) increases.

In the following examples we set *a*_1_(0) = 8 (to give the first PO a good chance to win the competition) and study how the variation of other parameters influences this probability.

#### **Example 4.**

In this example we vary the parameter *ν* of the function *f*. This parameter controls the steepness of the function *f* at 0. The results presented in Fig. 9 show that greater values of *ν* slightly increase the probability that the first PO will be the winner. More importantly, greater values of *ν* significantly decrease the probability that case (C) occurs.

#### **Example 5.**

This example is similar to Example 4 with the only difference that the range of the distribution of the natural frequencies of POs is 1.5, which prevents the possibility of stationary dynamics if the first PO is the winner. However, the system preserves the capability of WTA behavior with approximately the same efficiency as in Example 4. This is illustrated by Fig. 10. The probabilities of case (A) in both examples are similar. A significant decrease is only observed for *ν* = 20 from 76.3 in Example 4 to 69.3 in Example 6.

#### **Example 6.**

This example shows that the width of the range of the distribution of initial phases of POs plays a crucial role for determining the WTA regime. Figure 11 shows that the best results for WTA can be obtained if this range is small. The probability that the first PO wins the competition decreases rapidly if the width of the distribution increases. Also the probability of case (C) rapidly increases if the range of this distribution. exceeds 0.5*π*.

#### **Example 7.**

This example shows that the speed of adaptation of the amplitudes of POs *β* is important for successful implementation of the WTA regime. The best results are obtained when *β* is small (Fig. 12). This represents the fact that for proper functioning of the system the process of phase synchronization should be much faster than that of amplitude adaptation.

## Discussion

The objective of this paper was to suggest a WTA system whose functioning is based on oscillatory principles. As we have shown, the WTA can be realized in a system built from generalized phase oscillators with a central oscillator (CO). The WTA appears as a result of the competition between peripheral oscillators (POs) for synchronization with the CO. The amplification or inhibition of the activity of oscillators is a function of the phase relations between the CO and POs. In this respect our implementation of WTA differs from the traditional approach, where excitation or inhibition is directly conditioned by excitatory and inhibitory connections between the elements of the system. Our approach is consistent with the putative role of oscillatory processes in brain functions such as decision making and selective attention. It also opens the possibility of new hardware implementations of WTA.

Our results show that there are two different types of dynamics that can be associated with the WTA regime for the system in phase differences:

1. Stationary states, when there are constant relations between the phases of the CO and POs. In this case one of the POs (the winner) works inphase with the CO, while the difference between the phases of the CO and other POs is greater than *π*/2. A special case is represented by the system with identical natural frequencies of oscillators. In this case the “losers” work in anti-phase relative to the CO.
2. Non-stationary states. In this case one of the POs (the winner) works nearly coherently with the CO, while the set of POs is divided into two subsets. One subset contains the POs whose phases indefinitely move in the positive or negative direction relative to the phase of the CO. In the other subset the difference between the phase of a PO and the CO oscillates in a small range around a value greater than *π*/2.

According to Propositions 2 and 4, in the stationary case there cannot be more than one winning PO. This has been proved under two types of conditions. If the POs are identical, the statement is true for rather arbitrary functions of system (1)–(4). If POs have different natural frequencies, the statement is true at least for the functions specified by formulas (8)–(10).

Analytical analysis and numerical simulations show that the latter statement is also valid in the non-stationary case. The conditions for the existence of the stationary WTA regime are described by Lemmas 1–4 in the Methods section, Remark 3, and inequalities (35). The bifurcations that lead to the transformation of the WTA behavior from the stationary to non-stationary form are described by Propositions 5 and 6. The propositions describe a new type of bifurcation (saddle-node on invariant torus (SNIT) bifurcation) which has not been previously described in the literature.

Computer simulations under conditions (8)–(10) demonstrate that our WTA implementation is sensitive to the parameters of the system. The following conditions can increase the probability that the PO with the highest initial amplitude is the winner:

1. The highest initial amplitude is significantly greater than the initial amplitudes of the other POs.
2. Initial phases of POs are distributed in a small range around the point 0.
3. The interaction function *f* is steep at 0.
4. Slow adaptation of the amplitudes of POs relative to the process of phase-locking.

The second condition in this list is in line with the neurobiological role of phase reset in information processing in the brain^75^. The probability of an indefinite result of the competition can be reduced by the proper choice of parameter values.

## Methods

### Proofs of the main results

In this section we present the ideas of the proofs of the main results.

#### Stability of equilibria (Proof of Proposition 2)

For the functions *f*(*x*), *g*(*x*), *h*(*x*) satisfying conditions (5)–(7) (2n + 1)–dimensional system (16) has the Jacobian matrix

$${\bar{J}}_{2n+1}=(\begin{array}{ccccccc}-b{g}^{{\rm{^{\prime} }}}({\phi }_{1})-\frac{{a}_{1}}{n}{f}^{{\rm{^{\prime} }}}({\phi }_{1}) & \cdots  & -\frac{{a}_{n}}{n}{f}^{{\rm{^{\prime} }}}({\phi }_{n}) & -1 & -\frac{1}{n}f({\phi }_{1}) & \cdots  & -\frac{1}{n}f({\phi }_{n})\\ \vdots  & \ddots  & \vdots  & \vdots  & \vdots  & \cdots  & \vdots \\ -\frac{{a}_{1}}{n}{f}^{{\rm{^{\prime} }}}({\phi }_{1}) & \cdots  & -b{g}^{{\rm{^{\prime} }}}({\phi }_{n})-\frac{{a}_{n}}{n}{f}^{{\rm{^{\prime} }}}({\phi }_{n}) & -1 & -\frac{1}{n}f({\phi }_{1}) & \cdots  & -\frac{1}{n}f({\phi }_{n})\\ \frac{\alpha {a}_{1}}{n}{f}^{{\rm{^{\prime} }}}({\phi }_{1}) & \cdots  & \frac{\alpha {a}_{n}}{n}{f}^{{\rm{^{\prime} }}}({\phi }_{n}) & 0 & \frac{\alpha }{n}f({\phi }_{1}) & \cdots  & \frac{\alpha }{n}f({\phi }_{n})\\ \beta \gamma {h}^{{\rm{^{\prime} }}}({\phi }_{1}) & \cdots  & 0 & 0 & -\beta  & \cdots  & 0\\ \vdots  & \ddots  & \vdots  & \vdots  & \vdots  & \ddots  & \vdots \\ 0 & \cdots  & \beta \gamma {h}^{{\rm{^{\prime} }}}({\phi }_{2} & 0 & 0 & \cdots  & -\beta \end{array}).$$

In the block form this matrix is

$${\bar{J}}_{2n+1}=(\begin{array}{ccc}{A}_{n}+{B}_{n} & -{E}_{k,1} & {C}_{n}\\ {D}_{n} & 0 & {F}_{n}\\ {G}_{n} & {0}_{k,1} & -\beta {I}_{n}\end{array}),$$

where

$${A}_{n}=(\begin{array}{ccc}-\frac{{a}_{1}}{n}{f}^{{\rm{^{\prime} }}}({\phi }_{1}) & \cdots  & -\frac{{a}_{n}}{n}{f}^{{\rm{^{\prime} }}}({\phi }_{n})\\ \vdots  & \cdots  & \vdots \\ -\frac{{a}_{1}}{n}{f}^{{\rm{^{\prime} }}}({\phi }_{1}) & \cdots  & -\frac{\alpha {a}_{n}}{n}{f}^{{\rm{^{\prime} }}}({\phi }_{n})\end{array}),$$

$${B}_{n}={\rm{diag}}\{-bg^{\prime} ({\phi }_{1}),\ldots ,-bg^{\prime} ({\phi }_{n})\},$$

$${C}_{n}=(\begin{array}{ccc}-\frac{1}{n}f({\phi }_{1}) & \cdots  & -\frac{1}{n}f({\phi }_{n})\\ \vdots  & \cdots  & \vdots \\ -\frac{1}{n}f({\phi }_{1}) & \cdots  & -\frac{1}{n}f({\phi }_{n})\end{array}),$$

$${D}_{n}=(\frac{\alpha {a}_{1}}{n}{f}^{{\rm{^{\prime} }}}({\phi }_{1}),\ldots ,\frac{\alpha {a}_{n}}{n}{f}^{{\rm{^{\prime} }}}({\phi }_{n})),$$

$${F}_{n}=(-\frac{\alpha }{n}f({\phi }_{1}),\ldots ,-\frac{\alpha }{n}f({\phi }_{n})),$$

$${G}_{n}={\rm{diag}}\{\beta \gamma h^{\prime} ({\phi }_{1}),\ldots ,\beta \gamma h^{\prime} ({\phi }_{n})\},$$

where *I*_*n*_ = diag{1, …, 1} is the *n* × *n* identity matrix, *E*_*n*,*m*_ is the *n* × *m*-matrix of 1, 0_*n*,*m*_ is the *n* × *m*-matrix of 0.

Note that the stability of a fixed point does not depend on the common frequency *ω*.

Denote

$$p\mathop{=}\limits^{df}f^{\prime} \mathrm{(0),}\quad \mathop{{q}_{0}\,=\,}\limits^{df}g^{\prime} \mathrm{(0),}\quad {q}_{\pi }\mathop{=}\limits^{df}g^{\prime} (\pi \mathrm{)}.$$

Then for the fixed point *P*_*k*_ we have

$${A}_{n}({{\rm{\Phi }}}_{k})=(\begin{array}{cccccc}-\,\frac{{a}_{1}}{n}p & \cdots  & -\,\frac{{a}_{k}}{n}p & 0 & \cdots  & 0\\ \cdots  & \cdots  & \cdots  & \cdots  & \cdots  & \cdots \\ -\,\frac{{a}_{1}}{n}p & \cdots  & -\,\frac{\alpha {a}_{k}}{n}p & 0 & \cdots  & 0\end{array}),$$

$${B}_{n}({{\rm{\Phi }}}_{k})={\rm{diag}}\{\,-\,b,\ldots ,-b,\mathop{\underbrace{b,\ldots ,b}}\limits_{n-k}\},$$

$$C({{\rm{\Phi }}}_{k})={0}_{n,n},$$

$${D}_{n}({{\rm{\Phi }}}_{k})=(\frac{\alpha {a}_{1}}{n}p,\ldots ,\frac{\alpha {a}_{k}}{n}p,0,\ldots ,0),$$

$$F({{\rm{\Phi }}}_{k})={0}_{\mathrm{1,}n},\quad {G}_{n}({{\rm{\Phi }}}_{k})={0}_{n,n}.$$

The Jacobian matrix takes the form

$${\bar{J}}_{2n+1}=(\begin{array}{cc}{J}_{n+1}({{\rm{\Phi }}}_{k}) & 0\\ 0 & -\,\beta {I}_{n}\end{array}),$$

where

$${J}_{n+1}({{\rm{\Phi }}}_{k})=(\begin{array}{ccccccc}-b{q}_{0}-\frac{{a}_{1}}{n}p & \cdots  & -\frac{{a}_{k}}{n}p & 0 & \cdots  & 0 & -1\\ \vdots  & \ddots  & \vdots  & \vdots  & \cdots  & \vdots  & \vdots \\ -\frac{{a}_{1}}{n}p & \cdots  & -b{q}_{0}-\frac{{a}_{k}}{n}p & 0 & \cdots  & 0 & -1\\ -\frac{{a}_{1}}{n}p & \cdots  & -\frac{{a}_{k}}{n}p & -b{q}_{\pi } & \cdots  & 0 & -1\\ \vdots  & \cdots  & \vdots  & \cdots  & \ddots  & \cdots  & \vdots \\ -\frac{{a}_{1}}{n}p & \cdots  & -\frac{{a}_{k}}{n}p & 0 & \cdots  & -b{q}_{\pi } & -1\\ -\frac{\alpha {a}_{1}}{n}p & 0 & -\frac{\alpha {a}_{k}}{n}p & 0 & 0 & 0 & 0\end{array}).$$

The matrix ${\overline{J}}_{2n+1}({{\rm{\Phi }}}_{k})$ has *n* equal eigenvalues

$${\lambda }_{n+2}({{\rm{\Phi }}}_{k})=\cdots ={\lambda }_{2n+1}({{\rm{\Phi }}}_{k})=-\beta .$$

Other eigenvalues can be found as the eigenvalues of the (*n* + 1) × (*n* + 1)-block

$${J}_{n+1}({{\rm{\Phi }}}_{k},\omega ,{{\rm{\Psi }}}_{k})=(\begin{array}{ccccccc}-b{q}_{0}-\frac{(c+\gamma )}{n}p & \cdots  & -\frac{(c+\gamma )}{n}p & 0 & \cdots  & 0 & -1\\ \vdots  & \ddots  & \vdots  & \vdots  & \cdots  & \vdots  & \vdots \\ -\frac{(c+\gamma )}{n}p & \cdots  & -b{q}_{0}-\frac{(c+\gamma )}{n}p & 0 & \cdots  & 0 & -1\\ -\frac{(c+\gamma )}{n}p & \cdots  & -\frac{(c+\gamma )}{n}p & -b{q}_{\pi } & \cdots  & 0 & -1\\ \vdots  & \cdots  & \vdots  & \vdots  & \ddots  & \vdots  & \vdots \\ -\frac{(c+\gamma )}{n}p & \cdots  & -\frac{(c+\gamma )}{n}p & 0 & \cdots  & -b{q}_{\pi } & -1\\ \frac{\alpha (c+\gamma )}{n}p & \cdots  & \frac{\alpha (c+\gamma )}{n}p & 0 & \cdots  & 0 & 0\end{array})$$

$$=(\begin{array}{ccc}-b{q}_{0}{I}_{k}-\frac{(c+\gamma )}{n}p{E}_{k,k} & {0}_{k,n-k} & -{E}_{k,1}\\ -\frac{(c+\gamma )}{n}p{E}_{n-k,k} & -b{q}_{\pi }{I}_{n-k} & -{E}_{n-k,1}\\ \frac{\alpha (c+\gamma )}{n}p{E}_{1,k} & {0}_{1,n-k} & 0\end{array}).$$

These eigenvalues are

25 $$\begin{array}{c}{\lambda }_{1}({{\rm{\Phi }}}_{k})=\cdots ={\lambda }_{k-1}({{\rm{\Phi }}}_{k})=-b{g}^{{\rm{^{\prime} }}}(0),\\ {\lambda }_{k+2}({{\rm{\Phi }}}_{k})=\cdots ={\lambda }_{n+1}({{\rm{\Phi }}}_{k})=-b{g}^{{\rm{^{\prime} }}}(\pi ),\\ {\lambda }_{k,k+1}({{\rm{\Phi }}}_{k})=-\frac{1}{2n}({\sigma }_{k}+nb{g}^{{\rm{^{\prime} }}}(0)\pm \sqrt{{({\sigma }_{k}+nb{g}^{{\rm{^{\prime} }}}(0))}^{2}-4n\alpha {\sigma }_{k}}),\\ \qquad \qquad \qquad \qquad \qquad \qquad \qquad \qquad \qquad \qquad \qquad k=1,\ldots n,\end{array}$$

where $${\sigma }_{k}=f^{\prime} \mathrm{(0)}{\sum }_{j\mathrm{=1}}^{k}\,{a}_{j}$$, and

26 $${\lambda }_{1}({{\rm{\Phi }}}_{0})=\mathrm{0,}\quad {\lambda }_{2}({{\rm{\Phi }}}_{0})=\cdots ={\lambda }_{n+1}({{\rm{\Phi }}}_{0})=-bg^{\prime} (\pi \mathrm{)}.$$

Since *a*_*j*_ = *c* + *γ* for *j* = 1, …, *k*, then we have

$$\begin{array}{ccc}{\lambda }_{k,k+1}({{\rm{\Phi }}}_{k},\omega ,{{\rm{\Psi }}}_{k}) & = & -\displaystyle \frac{1}{2n}(k(c+\gamma ){f}^{{\rm{^{\prime} }}}(0)+nb{g}^{{\rm{^{\prime} }}}(0)\\  &  & \pm \sqrt{{(k(c+\gamma ){f}^{{\rm{^{\prime} }}}(0)+nb{g}^{{\rm{^{\prime} }}}(0))}^{2}-4nk\alpha (c+\gamma ){f}^{{\rm{^{\prime} }}}(0)}\,).\end{array}$$

Using these eigenvalues we can formulate stability conditions for fixed points of different types.

##### **Lemma 1.**

*The fixed point P*_0_ *of system* (16) *is stable along* 2*n directions and neutral along one direction if b* < 0.

The fixed point *P*_0_ corresponds to the antiphase regime of all POs relative to the CO (no-winner state). Linear neutrality of this point along one direction corresponds to the bifurcation line in the two–parametric bifurcation plane. The eigenvalues corresponding to *P*_0_ can also describe the dynamics when a continuous set of periodic orbits exists close to this point.

For the fixed point *P*_1_ we have

27 $$\begin{array}{ccc}{\lambda }_{3}({{\rm{\Phi }}}_{1}) & = & \cdots ={\lambda }_{n+1}({{\rm{\Phi }}}_{1})=-b{g}^{{\rm{^{\prime} }}}(\pi ),\\ {\lambda }_{1,2}({{\rm{\Phi }}}_{1}) & = & -\displaystyle \frac{1}{2n}({\sigma }_{1}+nb{g}^{{\rm{^{\prime} }}}(0)\pm \sqrt{{({\sigma }_{1}+nb{g}^{{\rm{^{\prime} }}}(0))}^{2}-4n\alpha {\sigma }_{1}}).\end{array}$$

It is stable when *bg*′(*π*) > 0 and Re(*λ*_1,2_(Φ_1_)) < 0:

$${\sigma }_{1}+nbg^{\prime} \mathrm{(0)}=(c+\gamma )f^{\prime} \mathrm{(0)}+nbg^{\prime} \mathrm{(0)} > \mathrm{0,}\quad n\alpha {\sigma }_{1}=n\alpha (c+\gamma )f^{\prime} \mathrm{(0)} > 0.$$

Using the assumption *g*′(*π*) < 0 we obtain.

##### **Lemma 2.**

*The fixed point P*_1_ *of system* (16) *is stable if*

$$b < \mathrm{0,}\quad (c+\gamma )f^{\prime} \mathrm{(0)}+nbg^{\prime} \mathrm{(0)} > \mathrm{0,}\quad \alpha (c+\gamma )f^{\prime} \mathrm{(0)} > 0.$$

Note that at least two eigenvalues of each fixed point *P*_*k*_, *k* = 2, …, *n* − 1, have opposite signs

$${\lambda }_{1}({{\rm{\Phi }}}_{k})=-bg^{\prime} \mathrm{(0)}=\frac{g^{\prime} \mathrm{(0)}}{g^{\prime} (\pi )}{\lambda }_{n+1}({{\rm{\Phi }}}_{k}),\quad k=2,\ldots n-1,$$

since as we assumed *g*′(0) > 0 and *g*′(*π*) < 0. Thus we have the following statement.

##### **Lemma 3.**

*The points P*_2_, …, *P*_*n*−1_ *are saddles for any values of the parameters*.

##### **Remark 3.**

If the condition *g*′(0)*g*′(*π*) < 0 is not fulfilled (for example, *g*(*x*) = sin(2*x*)) the points *P*_2_, …, *P*_*n*−1_ can be stable under the additional conditions Re(*λ*_*k*,*k*+1_(Φ_*k*_,*ω*,Ψ_*k*_)) < 0. However, in this case the function *g*(*x*) has additional intersections with the abscissa line *x* = 0, which leads to the appearance of additional fixed points (besides those mentioned in Proposition 1).

For the full synchronization associated with the fixed point *P*_*n*_ we have

$$\begin{array}{ccc}{\lambda }_{1}({{\rm{\Phi }}}_{n}) & = & \cdots ={\lambda }_{n-1}({{\rm{\Phi }}}_{n})=-b{g}^{{\rm{^{\prime} }}}(0),\\ {\lambda }_{n,n+1}({{\rm{\Phi }}}_{n}) & = & -\displaystyle \frac{1}{2n}({\sigma }_{n}+nb{g}^{{\rm{^{\prime} }}}(0)\pm \sqrt{{({\sigma }_{n}+nb{g}^{{\rm{^{\prime} }}}(0))}^{2}-4n\alpha {\sigma }_{n}}).\end{array}$$

##### **Lemma 4.**

*The point P*_*n*_ *is stable if n* ≥ 2 and

28 $$b > \mathrm{0,}\quad (c+\gamma )f^{\prime} \mathrm{(0)}+bg^{\prime} \mathrm{(0)} > \mathrm{0,}\quad \alpha (c+\gamma )f^{\prime} \mathrm{(0)} > 0.$$

In the case *n* = 1 the point *P*_*n*_ = *P*_1_ can be stable for both positive and negative values of the parameter *b* (in contrast to the case *n* ≥ 2). The condition for stability is *b* > −(*c* + *γ*)*f*′(0).

Summing up the obtained results we can formulate Proposition 2.

#### Non-identical natural frequencies, stationary solutions (the idea of the proof of Proposition 4)

In this subsection we obtain estimates of the coordinates of the point *Q*_*l*_ and derive conditions for its stability in the case when the functions *f*, *g*, *h* are specified as (8)–(10). We assume that the parameter *μ* of the function *h*(*x*) satisfies the inequality *μ* < *π*/2, so *h*(*x*) is not equal to zero in a relatively small interval (−*μ*, *μ*). The coordinates of *Q*_*l*_ are determined by the algebraic system

29 $${\omega }_{i}-{\omega }_{0}-b\,\sin ({\phi }_{i})-\frac{1}{n}\sum _{j\mathrm{=1}}^{n}{a}_{j}\,f({\phi }_{j})=\mathrm{0,}\quad i=1,\ldots ,n,$$

30 $$\sum _{j\mathrm{=1}}^{n}{a}_{j}f({\phi }_{j})=\mathrm{0,}$$

31 $$-{a}_{i}+c+\gamma h({\phi }_{i})=\mathrm{0,}\quad i=1,\ldots ,n,$$

with the additional suggestions that *φ*_*l*_ ∈ *D*_1_ = (−*π*/2, *π*/2), and *φ*_*i*_ ∈ *D*_2_ = (*π*/2, 3*π*/2) for *i* ≠ *l*.

From (31) we have *a*_*i*_ = *c*, *i* ≠ *l*. Equation (30) can be rewritten as

$$(c+\gamma h({\phi }_{l}))f({\phi }_{l})+c\sum _{i\ne l}f({\phi }_{i})=0.$$

Taking into account that

$$\mathop{{\rm{\max }}}\limits_{{{\mathscr{D}}}_{2}}\Vert f({\phi }_{i})\Vert =f(\pi \mathrm{/2})=\,\sin ({2}^{-\nu }\pi ),\quad i\ne l,$$

we can estimate the absolute value of *f*(*φ*_*l*_) as

$$|f({\phi }_{l})|\le (n-1)\frac{c\sin ({2}^{-\nu }\pi )}{(c+\gamma h({\phi }_{l}))}\le (n-1)\,\sin \,({2}^{-\nu }\pi ).$$

This implies that

32 $$|{\phi }_{l}|\le {f}^{-1}((n-\mathrm{1)}\,\sin ({2}^{-\nu }\pi ))\mathop{=}\limits^{df}{\phi }^{\ast },\quad {\phi }^{\ast }\in ({x}_{{\rm{\min }}},{x}_{{\rm{\max }}}).$$

In (32) the equality is only possible if *φ*_*i*_ = ±*π*/2, *i* ≠ *l*, that is if *ω*_*i*_ = *ω*_*l*_ ± *b*, *i* ≠ *l*. If the values of *σ* and *ν* are large, *φ*_*l*_ is very small. For example, if the parameter values are *n* = 100, *ν* = 20, then |*φ*_*l*_| < 10^−5^. In regular cases when the variables *φ*_*i*_ are distributed more or less uniformly around *π*, the variable *φ*_*l*_ is even much closer to zero.

Using inequality (32) we obtain a rough estimation for *ω*_0_ as

$$|{\omega }_{0}-{\omega }_{l}|\le |b|\,\sin |{\phi }^{\ast }|\le |b||{\phi }^{\ast }|.$$

The amplitude of the winning oscillator is estimated as

$$c+\gamma h({\phi }^{\ast })=c+\gamma {(\frac{{\mu }^{2}-{({\phi }^{\ast })}^{2}}{{\mu }^{2}})}^{\sigma }\le {a}_{l}\le c+\gamma .$$

From the expressions *ω*_*i*_ − *ω*_*l*_ − *b* sin *ϕ*_*i*_ ≈ 0 and taking into account that *φ*_*i*_ is close to *π* we estimate other coordinates of the fixed point as

$${\bar{\phi }}_{i}\approx \pi -\arcsin (\frac{{\omega }_{i}-{\omega }_{l}}{b}),\quad {\rm{w}}{\rm{h}}{\rm{e}}{\rm{n}}\quad |\frac{{\omega }_{i}-{\omega }_{l}}{b}|\le 1\quad {\rm{f}}{\rm{o}}{\rm{r}}\,{\rm{a}}{\rm{n}}{\rm{y}}\quad i\ne l.$$

Thus the equilibrium *Q*_*l*_ can be approximately represented as

33 $$({\overline{\phi }}_{1},\ldots ,{\overline{\phi }}_{l-1},\,0,{\overline{\phi }}_{l+1},\ldots ,{\overline{\phi }}_{n},{\omega }_{l},c,\ldots ,c,\mathop{\underbrace{c+\gamma }}\limits_{n+1+l},c,\ldots c).$$

Let us derive conditions for the stability of the fixed point *Q*_*l*_ in the case when *ν* is large enough. In this case *φ*_*l*_ ≈ 0, therefore *f*(*φ*_*l*_) ≈ 0, *f*′(*φ*_*l*_) ≈ *ν*. The values of *f*(*φ*_*i*_) and *f*′(*φ*_*i*_), *i* ≠ *l*, are both very close to zero in some range around the point *π*. For example, both ${{\rm{\max }}}_{{D}_{2}}|f({\phi }_{i})|=f(\pi \mathrm{/2)}$ and ${{\rm{\max }}}_{{D}_{2}}|f^{\prime} ({\phi }_{i})|=-f^{\prime} (\pi \mathrm{/2)}$ are less than 10^−5^ in the case *ν* = 20, therefore for *l* = 1 the Jacobian at the point *Q*_1_ is approximately

34 $${\overline{J}}_{2n+1}({Q}_{1})\approx {\rm{diag}}\{{J}_{n+1}({Q}_{1}),-\beta {I}_{n}\},$$

where *J*_*n*+1_(*Q*_1_) has the form

$${J}_{n+1}({Q}_{1})=(\begin{array}{ccccc}-b-\frac{c+\gamma }{n}\nu  & 0 & \cdots  & 0 & -1\\ -\frac{c+\gamma }{n}\nu  & -b\cos {\bar{\phi }}_{2} & \cdots  & 0 & -1\\ \vdots  & \vdots  & \ddots  & \vdots  & \vdots \\ -\frac{c+\gamma }{n}\nu  & 0 & \cdots  & -b\cos {\bar{\phi }}_{n} & -1\\ \frac{\alpha (c+\gamma )}{n}\nu  & 0 & \cdots  & 0 & 0\end{array}).$$

The Jacobian has a similar structure for an arbitrary *l* with the only difference that the *l*-th column is filled like the first one in the presented matrix. Now we can calculate the eigenvalues of the fixed point with a high precision:

$${\lambda }_{l,n+1}=-\frac{1}{2n}((c+\gamma )\nu +nb\pm \sqrt{{((c+\gamma )\nu +nb)}^{2}-4n\alpha (c+\gamma )}),$$

$${\lambda }_{i}=-b\,\cos ({\bar{\phi }}_{i}),\quad i=1,\ldots ,n,\quad i\ne l,$$

$${\lambda }_{i}=-\beta ,\quad i=n+2,\ldots ,\,2n+1.$$

Thus *Q*_*l*_ is stable when

35 $$(c+\gamma )\nu +nb\mathrm{ > 0,}\quad n\alpha (c+\gamma )\nu  > \mathrm{0,}\quad b\,\cos ({\phi }_{i}) > \mathrm{0,}\quad i\ne l.$$

Since it is assumed that *b* < 0, the latter inequality is valid when ${\bar{\phi }}_{i}$ is inside the interval ${{\mathscr{D}}}_{2}$.

Thus, we have shown that the phase coordinate *φ*_*l*_ (the difference between the winning PO and the CO) of the fixed point *Q*_*l*_ is very close to zero while phase differences of loser POs ${\phi }_{i}$, *i* ≠ *l*, are located around *π*. At the same time, the amplitude of the winning PO is approximately *c* + *γ* while the amplitudes of other POs are close to *c*. We have also shown that the current frequency *ω*_0_(*t*) of the CO tends to the natural frequency *ω*_*l*_ of the winner. Using these consideration we can formulate Proposition 4.

#### Non-identical natural frequencies, non-stationary solutions (the idea of the proof of Propositions 5, 6)

As has been shown above, there are *n* different equilibria *Q*_*l*_, *l* = 1, …, *n*, with coordinates that are approximately represented by formula (21). Each coordinate ${\bar{\phi }}_{i}$, *i* ≠ *l*, in this formula is one of the two solutions of the equation *b*sin*ϕ*_*i*_ = *ω*_*i*_ − *ω*_*l*_, namely the one that is closer to *π*. The necessary condition for the existence of such fixed points is max_*i*≠*l*_|(*ω*_*l*_ − *ω*_*i*_)/*b*| ≤ 1. One can check (in a similar way that has been used for the stable fixed point *Q*_*l*_) that there exist *n* − 1 other fixed points *S*_*l*,*j*_, *j* ≠ *l*, which have the same coordinates as *Q*_*l*_ except one coordinate ${\phi }_{j}={\tilde{\phi }}_{j}=\pi -{\bar{\phi }}_{j}\approx \arcsin (({\omega }_{j}-{\omega }_{l})/b)$. The point *S*_*l*,*j*_ has the same Jacobian (34) as *Q*_*l*_ but with ${\tilde{\phi }}_{j}$ instead of ${\bar{\phi }}_{j}$. In the conditions of stability each fixed point *S*_*l*,*j*_ has one positive eigenvalue ${\lambda }_{j}=-b\,\cos ({\tilde{\phi }}_{j})$. This means that *S*_*l*,*j*_ is a saddle point that belongs to a 1–dimensional unstable invariant manifold *W* ^*u*^(*S*_*l*,*j*_). The eigenvector corresponding to the eigenvalue *λ*_*j*_ belongs to the coordinate line of the variable *φ*_*j*_. This means that both branches of *W* ^*u*^(*S*_*l*,*j*_) have the same coordinate as *S*_*l*,*j*_ (and *Q*_*l*_) except *φ*_*j*_ and these branches spread along the variable *φ*_*j*_ (at least locally) (Fig. 2(a)). The divergence in the transversal direction to *W* ^*u*^(*S*_*l*,*j*_) along *φ*_*j*_ is negative (the Jacobian shows that *W* ^*u*^(*S*_*l*,*j*_) is stable in the transversal direction), which implies that both branches of *W* ^*u*^(*S*_*l*,*j*_) reach the stable point *Q*_*l*_ from two opposite sides along *φ*_*j*_, forming a locked circle. The local saddle–node bifurcation of *Q*_*l*_ and *S*_*l*,*j*_ (Fig. 2(b)) is simultaneously a global (SN) bifurcation on the invariant circle, (SNIC bifurcation), and it leads to the appearance of a stable limit cycle *LC*_*l*_ (Fig. 2(c)). This bifurcation can happen if only |(*ω*_*l*_ − *ω*_*j*_)/*b*| = 1 at the point *φ*_*j*_ = ±*π*/2. Due to (24) SNIC bifurcations and disappearance of *Q*_*l*_ can only happen if *j* = 1 or *j* = *n*. It is obvious that the first (in the sense of the continuous movement of *ω*_*i*_ away from *ω*_*l*_) such bifurcation occurs when *ω*_1_ − *ω*_*n*_ = |*b*| and *l* = 1 or *l* = *n*. This bifurcation simultaneously happens with both of the two POs, the first and the last in numeration (24).

Thus, we have two simultaneous SNIC bifurcations with the points *Q*_1_, *S*_1,*n*_ and *Q*_*n*_, *S*_*n*,1_, where *Q*_1_ is the winning PO with the largest frequency *ω*_1_ and ${S}_{1}\,:={S}_{\mathrm{1,}n}$ is the corresponding saddle with the unstable manifold *W* ^*u*^(*S*_1_) along the phase variable *φ*_*n*_, (the same for *Q*_*n*_ and *W* ^*u*^(*S*_*n*_), where ${S}_{n}:={S}_{n\mathrm{,1}}$). As a result of the bifurcations, we have stable limit cycles *LC*_1_, *LC*_*n*_, where each of the cycles has one unbounded phase variable *φ*_*n*_ and *φ*_1_, respectively. Summing up the above considerations we can formulate Proposition 5.

The cycle *LC*_1_ is non-homologous to the zero periodic orbit on the torus ${{\mathbb{T}}}^{n}$ in phase space, the variable *φ*_*n*_ of the cycle is unbounded while all other phase variables are bounded (the same is true for *LC*_*n*_ and *φ*_1_). SNIC bifurcations with the fixed points *Q*_1_, *Q*_*n*_ are not only possible ones. Other SNIC bifurcations at the point *Q*_*l*_ occur when the distance *ω*_*l*_ − *ω*_*n*_ (or the distance *ω*_1_ − *ω*_*l*_) is equal to |*b*|, while the distance *ω*_1_ − *ω*_*l*_ (respectively, *ω*_*l*_ − *ω*_*n*_) is smaller than |*b*|. Such bifurcations lead to the appearance of new cycles *LC*_*l*_, *l* = 2, …, *n* − 1, and they usually happen simultaneously with more complex bifurcation of the existing cycles *LC*_1_, *LC*_*n*_ which we will describe later.

Besides limit cycles with one running variable *φ*_*i*_ (*i* ≠ *l*), the non-stationary WTA regime can be associated with more complex solutions when *n* ≥ 3 and there are several POs (more then one) whose variables *φ*_*i*_ are boundlessly running in the positive or negative direction. Let us describe how such regimes may appear.

Consider a 2–dimensional hyperplane with free variables *φ*_*j*_, *φ*_*m*_ and all other variables being the constant coordinates of the fixed point *Q*_*l*_ (Fig. 2(a)). In this hyperplane the point *Q*_*l*_ has the coordinates $({\bar{\phi }}_{j},{\bar{\phi }}_{m})$ and the saddle *S*_*l*,*j*_ has the coordinates $(\pi -{\bar{\phi }}_{j},{\bar{\phi }}_{m})$. The SNIC bifurcation of *Q*_*l*_ and *S*_*l*,*j*_ leads to the emergence of a saddle-node point *SN*_*l*,*j*_ (Fig. 2(b)) and then to a stable limit cycle *LC*_*l*_ (Fig. 2(c)). According to the *m*–th equation (23), the system has also two additional fixed points *S*_*l*,*m*_ belonging to the 1–dimensional manifold *W* ^*u*^(*S*_*l*,*m*_) and the saddle *S*_*l*,*j*,*m*_ belonging to the 2–dimensional unstable manifold *W* ^*uu*^(*S*_*l*,*j*,*m*_) (*S*_*l*,*j*,*m*_ is an unstable node on the 2-dimensional plane $({\phi }_{j},{\phi }_{m})\in {{\mathbb{T}}}_{l}^{2}$). The points *S*_*l*,*m*_ and *S*_*l*,*j*,*m*_ have a common 1–dimensional invariant manifold which is stable for the first point and unstable for the second one. In other worlds, these points lie on the circle formed by two branches of the manifold. The SNIC bifurcation of *S*_*l*,*m*_ and *S*_*l*,*j*,*m*_ leads to the appearance of a saddle-node point *SN*_*l*,*j*,*m*_ (Fig. 2(b)) and then to a saddle limit cycle *SC*_*l*_ (Fig. 2(c)) with one unstable invariant manifold, spreading along the variable *φ*_*m*_. Two SNIC bifurcations and the appearance of *LC*_*l*_ and *SC*_*l*_ happen almost simultaneously (Fig. 2(a–c)). In this case we have stable and unstable limit cycles on the torus ${{\mathbb{T}}}_{l}^{2}.$ In the case of the non–simultaneous SNIC bifurcations we have two variants of Cherry flows^68,69,74,76^ on the torus ${{\mathbb{T}}}_{l}^{2}$: 1) a stable cycle *LC*_*l*_ and two fixed points *S*_*l*,*m*_, *S*_*l*,*j*,*m*_ or 2) a saddle (unstable on ${{\mathbb{T}}}_{l}^{2}$) limit cycle *SC*_*l*_ and two fixed points *Q*_*l*_, *S*_*l*,*m*_. Note that the manifold ${ {\mathcal M} }_{l}^{2}$ of system (12)–(14) (that is represented here by the torus ${{\mathbb{T}}}_{l}^{2}$) is a 2–dimensional unstable invariant manifold of the saddle cycle *SC*_*l*_ which is stable in the transversal 2*n* − 1 directions in global (2*n* + 1)–dimensional phase space ${{\mathbb{T}}}^{n}\times {\mathbb{R}}\times {{\mathbb{R}}}^{n}$ (when *SC*_*l*_ exists).

The *saddle*–*node bifurcation of LC*_*l*_ *and SC*_*l*_ *on the invariant torus* ${{\mathbb{T}}}_{l}^{2}$ (SNIT) is similar to the SNIC bifurcations considered above (one can also call this bifurcation *a saddle*–*node of periodic orbits with global reinjection*). Locally, it is a saddle–node (fold) bifurcation of the stable cycle *LC*_*l*_ and the saddle cycle *SC*_*l*_ (which happens along the variable *φ*_*m*_) that leads to the transitivity of trajectories through the torus. As a result of the SNIT bifurcation, we obtain first saddle-node limit cycle *SNC*_*l*_ (Fig. 2(d)) and then a stable (in transversal direction) limit torus that is denoted as $L{T}_{l}^{2}$ (Fig. 2(e)). The described SNIT bifurcation is a global bifurcation on the torus ${{\mathbb{T}}}_{l}^{2}$ (as SNIC is a global bifurcation on a circle) and it leads to transitivity of the trajectories on the whole 2D torus. We note that the transitivity on the whole two-torus is true only for the fat Cantor set of parameter values with irrational winding ratio. The bifurcation occurs when the phase difference |*ω*_*l*_ − *ω*_*m*_| reaches the value |*b*| but |*ω*_*l*_ − *ω*_*j*_| ≥ |*b*| and |*ω*_*l*_ − *ω*_*i*_| < |*b*| for *i* ≠ *l*, *i* ≠ *j*, *i* ≠ *m*. We already mentioned that if the range of the natural frequencies of POs increases the first two SNIC bifurcations occur simultaneously when the 1-st or the *n*-th PO becomes the winner. This leads to the appearance of the limit cycles *LC*_1_and *LC*_*n*_. One can check that enlarging further the frequency distribution we obtain a SNIT bifurcation simultaneously with a SNIC bifurcation. As a result of such simultaneous bifurcations, the pair of $L{T}_{1}^{2}$ and *LC*_*n*−1_ or the pair of $L{T}_{n}^{2}$ and *LC*_2_ appears. The emergence of other limit tori is associated with more complex bifurcations of a similar type.

In the same way it is possible to show that there exists a saddle torus $S{T}_{l}^{2}$ that appears as a result of a SNIT bifurcation of two saddle limit cycles with 1-dimensional and 2-dimensional unstable manifolds. Two simultaneous bifurcations of the appearance of stable and saddle 2D invariant tori $L{T}_{l}^{2}$ and $S{T}_{l}^{2}$ are shown in Fig. 3(c–e). The tori $L{T}_{l}^{2}$ and $S{T}_{l}^{2}$ belong to the 3-dimensional torus ${{\mathbb{T}}}_{l}^{3}$ which is an invariant manifold of the $S{T}_{l}^{2}.$ As in the case of lower dimensions, the fold bifurcation of two invariant tori $L{T}_{l}^{2}$ and $S{T}_{l}^{2}$ leads to the emergence of the saddle-node torus $SN{T}_{l}^{2}$ (Fig. 3(f)), then to its disappearance and possibly to the transition of all trajectories through the whole 3-dimensional manifold. Thus, globally we have a new SNIT bifurcation of the next level which leads to the emergence of a new stable torus $L{T}_{l}^{3}$ in the global phase space which is passable for trajectories of the system. This torus appears when two frequency differences |*ω*_*l*_ − *ω*_*i*_| are greater than |*b*|, one such difference is equal to |*b*| and the other *n* − 3 differences are lower than |*b*|. This torus exists when for 3 values of the index *i* the inequality |*ω*_*l*_ − *ω*_*i*_| > |*b*| takes place, while for other values of the index *i* the inequality |*ω*_*l*_ − *ω*_*i*_| < |*b*| is valid.

Using similar arguments, one can describe the SNIT bifurcation of $L{T}_{l}^{m-1}$ and $S{T}_{l}^{m-1}$ that leads to the appearance of the stable tori $L{T}_{l}^{m}$ for any *m* = 1, …, *n* − 1. According to our notation $L{T}_{l}^{1}=L{C}_{l}$, $S{T}_{l}^{1}=S{C}_{l}$, $L{T}_{l}^{0}=Q$, $S{T}_{l}^{0}={S}_{l}$. The torus $L{T}_{l}^{m}$ exists when |(*ω*_*l*_ − *ω*_*i*_)/*b*| > 1 for *m* different values of the index *i* ≠ *l* and |(*ω*_*l*_ − *ω*_*i*_)/*b*| < 1 for the rest *n* − *m* − 1 values of the index *i*. Proposition 6 sums up these considerations.
